# The L-DBF vaccine cross protects mice against different *Shigella* serotypes after prior exposure to the pathogen

**DOI:** 10.1128/spectrum.00062-23

**Published:** 2023-10-03

**Authors:** Ti Lu, Debaki R. Howlader, Sayan Das, Zackary K. Dietz, Aaron C. Nagel, Sean K. Whittier, William D. Picking, Wendy L. Picking

**Affiliations:** 1 Department of Pharmaceutical Chemistry, University of Kansas, Lawrence, Kansas, USA; 2 Hafion, Inc, Lawrence, Kansas, USA; Post Graduate Institute of Medical Education and Research, Chandigarh, India

**Keywords:** shigellosis, pre-exposure, IpaB, IpaD, vaccine, IL-17, IFN-γ, Il-6

## Abstract

Shigellosis (bacillary dysentery) is a severe diarrheal disease caused by members of the genus *Shigella*, which results in 90 million cases annually around the world. The *Shigella* type III secretion system (T3SS) is a specialized secretion system that is the primary virulence factor it uses to infect the colonic mucosa. The type III secretion apparatus (T3SA) proteins IpaB and IpaD, as well as the genetic fusion, DBF, have been demonstrated to protect mice from *Shigella* spp. infection in a lethal pulmonary model. In a previous study, we fused LTA1, the active moiety of lethal toxin from enterotoxigenic *Escherichia coli* to DBF to produce a self-adjuvanting vaccine candidate L-DBF, which cross-protected mice against four serotypes of *Shigella flexneri* and *Shigella sonnei*. Here, we exposed mice with one or two sublethal doses of *S. flexneri* 2a to identify whether the immune response induced by L-DBF in the host would be affected by prior infection by homologous or heterologous *Shigella* serotypes. We demonstrate that pre-infection with two sublethal doses of *S. flexneri* 2a did not elicit cross-protection against *S. sonnei*, while vaccination with L-DBF did. Our results indicate that L-DBF is a feasible vaccine candidate that offers cross-protection against *Shigella*’s different serotypes even after prior exposure to the pathogen. This work provides a proof of concept that a novel subunit vaccine can not only protect a naïve host from *Shigella* challenge, but also can protect against challenge after prior infection by the same or different *Shigella* serotypes.

**IMPORTANCE:**

Shigellosis is endemic to low- and middle-income regions of the world where children are especially vulnerable. In many cases, there are pre-existing antibodies in the local population and the effect of prior exposure should be considered in the development and testing of vaccines against *Shigella* infection. Our study shows that L-DBF-induced immune responses are not adversely affected by prior exposure to this pathogen. Moreover, somewhat different cytokine profiles were observed in the lungs of vaccinated mice not having been exposed to *Shigella*, suggesting that the immune responses elicited by *Shigella* infection and L-DBF vaccination follow different pathways.

## INTRODUCTION

Shigellosis is a severe gastrointestinal disease that is estimated annually to affect 90 million people globally, with 164,000 deaths reported each year (1). Shigellosis disproportionately affects low-income regions of the world where potable water and proper sanitation are lacking. Children are particularly vulnerable, with mortality and morbidity rates highest among those under the age of five (1). Survivors often suffer from impaired growth due to malnutrition, which is exacerbated by repeated episodes of infection (2). *Shigella* spp. also cause diarrhea in travelers and military personnel in countries with low, middle incomes (LMIC) (3 – 5) or even high incomes (6 – 8). The *Shigella* species include *S. flexneri*, *S. sonnei*, *S. dysenteriae*, and *S. boydii*, which are further divided into more than 50 serotypes that offer little or no cross-protective immunity (4, 9). *S. flexneri* is the primary species of endemic diarrhea in developing regions where there is limited access to a hygienic resource, whereas *S. sonnei* is the primary serotype in more developed regions (10, 11). The symptoms of shigellosis include watery diarrhea, bloody dysentery, fever, intestinal cramps, and vomiting (1). Despite significant reductions in frequency due to improved sanitation, the rise of antimicrobial resistance in *Shigella* prompts the development of a safe and effective vaccine against this pathogen (9). While there are several vaccine candidates currently being developed, no licensed vaccine is available yet (12).

Resembling a syringe and needle, the type III secretion system (T3SS) apparatus (T3SA) is an energized nanomachine that is used by the pathogen to inject virulence effector proteins into host cells to manipulate the host cell machinery for the benefit of the bacterium (13). The *Shigella* T3SA tip protein IpaD localizes to the T3SA needle tip and is required for secretion control and virulence (14). IpaD recognizes extracellular signals to trigger the surface localization of IpaB, the first *Shigella* T3SA translocator protein, to dock with IpaD at the needle tip (15). Because IpaD and IpaB are highly conserved among *Shigella* species, broadly protective subunit vaccines against *Shigella* could be developed using these proteins (16).

The intranasal (IN) administration of IpaD and IpaB with the mucosal adjuvant double-mutant heat labile toxin (dmLT) from enterotoxigenic *Escherichia coli* provides protection in mice against challenge with both homologous and heterologous strains of *S. flexneri* and *S. sonnei* (16). By producing an IpaD-IpaB genetic fusion, called DBF, we were able to reduce production costs. When delivered IN, DBF admixed with dmLT induced immune responses similar to those stimulated by IpaD, IpaB, and dmLT (17). This formulation also provided cross-protection against *S. flexneri, S. sonnei,* and *S. dysenteriae*. Moreover, by genetically fusing LTA1, the active moiety of dmLT, to the N-terminus of DBF, we produced L-DBF and reduced the risk of the potential side effects of dmLT (18) while further reducing production costs (19). L-DBF was found to elicit significant levels of IL-17 and IFN-γ in vaccinated mice, and was effective in protecting them against a lethal challenge of *S. flexneri* 2a. Importantly, L-DBF also conferred cross-protection against lethal heterologous challenges with *S. flexneri* 3a, 1b, and 6 and *S. sonnei* (19). Both Th1 and Th17 responses are known to be important for protection against an *S. flexneri* infection (19, 20).

The recent Global Enteric Multicenter Study (GEMS) found that 24% of shigellosis cases were caused by *S. sonnei* and 66% by *S. flexneri* in low-income countries (21), while *S. sonnei* is responsible for a large majority of cases in middle- and high-income countries (21). Thus, a broadly protective vaccine must cover not only a naïve population but also a population that may have already experienced shigellosis. In this study, we examine the potential of L-DBF to elicit protective immunity in mice that have been pre-exposed to *S. flexneri*. We found that one or two sublethal exposures to *S. flexneri* 2a does not affect the subsequent immune responses in mice vaccinated with L-DBF. Mice pre-exposed to *S. flexneri* 2a produced bactericidal antibodies against *S. flexneri* 2a with the target of those antibodies being mainly LPS with a lower level of bacteriocidal anti-IpaB and anti-IpaD antibodies, however, these antibodies were not long lasting. In contrast, if the mice were vaccinated with L-DBF, the anti-IpaB and anti-IpaD antibodies were much more long lived. Moreover, mice pre-exposed with *S. flexneri* 2a were unable to survive a subsequent *S. sonnei* challenge, while pre-exposed mice subsequently vaccinated with L-DBF did survive *S. sonnei* challenge. Since it can protect against heterologous *Shigella* spp. serotypes, regardless of prior pathogen exposure, L-DBF has the potential to be a broadly protective vaccine against shigellosis in sporadic and endemic areas of the world.

## RESULTS

### A single pre-exposure to *S. flexneri* 2a is not sufficient to protect mice from a homologous challenge while L-DBF does protect against homologous or heterologous challenge

It has been previously demonstrated that a single *S. flexneri* 2a exposure is not sufficient to elicit protective immunity in mice from a subsequent challenge, even against the homologous serotype (22). It’s unclear, however, whether a prior infection might alter the protective immunity elicited by L-DBF. Therefore, we wanted to examine the immune response induced by L-DBF in naïve mice alongside those previously exposed to *S. flexneri*. Thus, half of a group of mice were pre-exposed with 6 × 10^4^ CFU of *S. flexneri* 2a on day −60. On day 0, half of the mice from the pre-exposed or “treatment” group (T) and half from the non-exposed or “no treatment” group (NT) were vaccinated (V) with 25 µg L-DBF IN (Table 1). The remaining mice from each group were administered PBS as negative controls (not vaccinated or NV). The kinetics of the antigen specific IgG and IgA response showed that all vaccinated mice had higher antibody titers than unvaccinated mice (Fig. S1). Pre-exposed mice (T) exhibited low antibody titers at the end of the pre-exposure period as compared to the high titers of anti-IpaD and anti-IpaB post-L-DBF vaccination (NT-V) (Fig. S1A and B). However, anti-IpaB and anti-IpaD IgG and IgA titers increased to comparable levels after the pre-exposed mice were immunized with L-DBF (T-V). Very low titers of anti-LPS IgG in the pre-exposure groups were also observed, which decreased over time (Fig. S1C). Although some anti-LPS IgG and IgA were detected in NT-V, they are below our cut-off. These four groups of mice (NT-NV, NT-V, T-NV, and T-V) were challenged on day 56 with a potentially lethal dose of *S. flexneri* 2a (6 × 10^6^ CFU), *S. flexneri* 1b (4 × 10^6^ CFU), or *S. sonnei* (1 × 10^6^ CFU). Fewer than 30% of the unvaccinated mice (NT-NV, T-NV) survived the challenges including those treated groups that were challenged with the homologous *S. flexneri* 2a strain. In contrast, >90% of those mice vaccinated with L-DBF (NT-V, T-V) survived the *Shigella* spp. challenge, regardless of challenge species. Table 1 demonstrates the broadly protective capacity of L-DBF. Similar results were seen in the weight loss curves (Fig. S2). Those mice that were vaccinated with L-DBF (NT-V and T-V) consistently demonstrated an earlier initiation of weight gain during recovery from the challenge than did the small number of unvaccinated mice (NT-NV and T-NV) that survived the challenge. We have found that weight gain is a strong early indicator of survival.

**TABLE 1:** Protective efficacy of L-DBF against *Shigella* spp. serotypes following a single pre-exposure^*a*^

Set	Grp	Pre-exposure treatment (T)	Vaccination (V)	Homologous challenge	Heterologous challenge	
		*S. flexneri* 2a	25 µg L-DBF	*S. flexneri* 2a	*S. flexneri* 1b	*S. sonnei* 53G
A	1	NT	NV	0%	–	–
	2	NT	NV	–	20%	–
	3	NT	NV	–	–	20%
B	4	NT	V	90%	–	–
	5	NT	V	–	100%	–
	6	NT	V	–	–	90%
C	7	T	NV	10%	–	–
	8	T	NV	–	30%	–
	9	T	NV	–	–	30%
D	10	T	V	100%	–	–
	11	T	V	–	100%	–
	12	T	V	–	–	90%

^*a*^ The pre-exposure groups were treated as follows: Sets A and B were not pre-exposed to *S. flexneri* 2a (not treated or NT). Sets C and D were pre-exposed to *S. flexneri* 2a (T). On day 0, sets A and C were administered PBS (not vaccinated or NV). Sets B and D were vaccinated with L-DBF (V). On day 56, all mice were challenged with results given as percent survival. –, not applicable.

### Two *S. flexneri* 2a pre-exposures does protect against a subsequent homologous challenge and does not affect the homologous or heterologous protection elicited by L-DBF

While we felt it was important to show that pre-exposure to *Shigella* did not reduce L-DBF efficacy, it is equally important to show that multiple pre-exposure episodes that do provide subsequent protective immunity against a given serotype do not adversely affect the L-DBF vaccination. It has been shown previously that a single *Shigella* infection does not protect against a subsequent homologous challenge; however, two such infections do provide protection against a third challenge by the same serotype (22). We, therefore, exposed mice to a lower dose (10^5^ bacteria), allowed them to fully recover and then exposed them to a second larger (6 × 10^5^) and allowed them to again recover. We then vaccinated with L-DBF to determine the effect of the double exposure on the resulting immune response.

**Fig 1:**
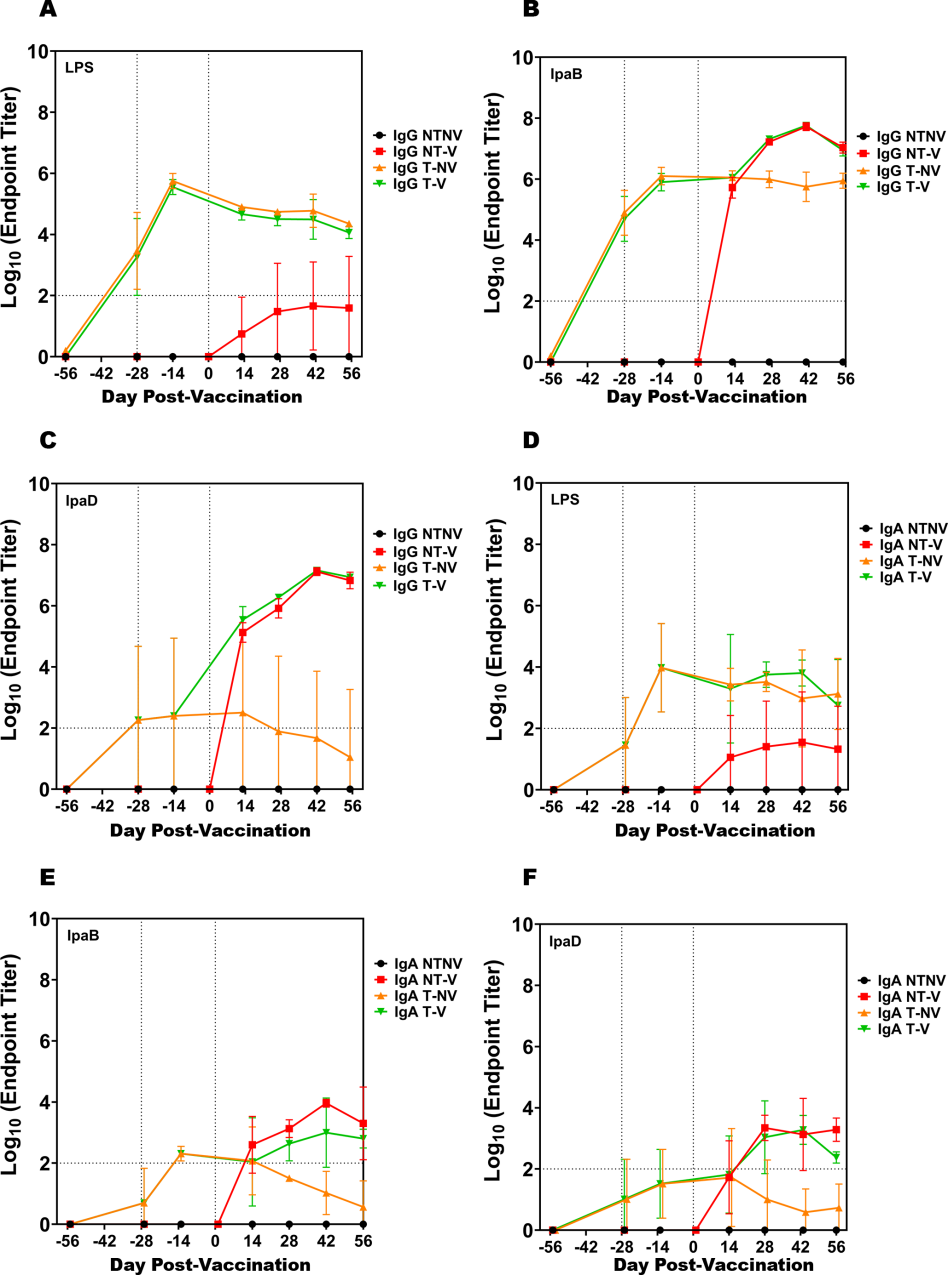
Kinetics of serum IgG and fecal IgA titers from mice exposed twice to *S. flexneri* 2a. Half of the mice were infected (treated or T or untreated NT) with a sublethal dose of *S. flexneri* 2a on days −56 and −28. Half of each group of mice were then vaccinated IN three times (days 0, 14, and 28) (vaccinated or V or not vaccinated NV). Blood and fecal samples were collected and measured titers for anti-LPS (A and D) or anti-IpaB (B and E) or anti-IpaD (C and F) IgG and IgA by ELISA. More than 40% of serum samples from the T-NV group did not have detectable titers of anti-IpaD during all the timepoints. More than 50% of the serum samples from the NT-V group did not have detectable titers of anti-LPS during all the timepoints. The individual titers are represented as EU mL^−1^. Each point represents the mean of each group (*n* = 10/group).

For these experiments, half of the mice were pre-exposed to *S. flexneri* 2a on days −56 and −28 with 82% of the mice surviving both pre-exposures. On day 0, half of the mice from the pre-exposed treated group (T) and half from the non-exposed or not treated group (NT) were vaccinated with 25 µg L-DBF IN (V). The remaining mice from each group were administered PBS to serve as not vaccinated controls (NV). As compared to a single pre-exposure, anti-LPS IgG titers after two-pre-exposures were approximately the same at day 0 (T-NV, T-V); however, the durability of the IgG response was better with a second pre-exposure (Fig. 1A; Fig. S1C). In contrast, while class switching (in terms of anti-LPS IgA titers) was considered at background levels (log = 2) after a single pre-exposure, they were significantly higher than background levels after two pre-exposures. Furthermore, these titers were durable since they were maintained above background at day 56 (Fig. 1A). All mice vaccinated with L-DBF elicited higher antibody titers than non-vaccinated mice (Fig. 1). The maximal anti-IpaB IgG titers were comparable regardless of number of pre-exposures (Fig. 1B; Fig. S1A). Similarly, after the prime vaccination (as detected in day 14 samples), T-NV and T-V mice elicited comparable anti-IpaB IgG, IgA and IgM titers to those of NT-V (Fig. 1B; Fig. S3A) with the anti-IpaB IgM titers of the NT-V being slightly lower. After the first boost on day 14, the anti-IpaB IgG and IgA titers declined in the T-NV group but showed an increase in the T-V group compared to the high titers seen for the NT-V mice. Thus, anti-IpaB antibody responses were similarly primed regardless of the number of pre-exposures, albeit the response was faster upon vaccination for the mice with two pre-exposures, reaching maximal titers after the first boost on day 14 rather than with the second boost at day 28 as seen for the single pre-exposure mice. While the anti-IpaB and anti-IpaD kinetics of IgG and IgA titers were similar after a single pre-exposure, the anti-IpaD IgG and IgA kinetics after the two dose *S. flexneri* 2a pre-exposure were distinct (Fig. 1C; Fig. S3A). Anti-IpaD IgM, IgG, and IgA would be considered below baseline for all four groups prior to vaccination (day −14) (Fig. 1C; Fig. S3A). In contrast, at day 14 the T-V group had a significant increase in anti-IpaD IgM and IgG and an increase above baseline for IgA while the T-NV mice remained at pre-vaccination levels and continued to decrease thereafter. Notably, all mice tested showed anti-IpaB antibodies, but only 60% of serum from the T-NV mice contained detectable anti-IpaD antibodies, which led to the large standard deviations. This could be due to the difference in immunogenicity between these two subunits. Based on the anti-IpaD antibodies levels elicited after the prime and first boost for the NT-V mice, it appears that the second pre-exposure primed the T-V immune response to IpaD and the vaccination at day 0 provided a boost that increased antibody titers. Maximal titers for both NT-V and T-V groups were reached after the third L-DBF vaccination.

The four groups of mice were challenged on day 56 with lethal doses of *S. flexneri* 2a (1.5 × 10^6^ CFU/mouse), *S. flexneri* 1b (1.5 × 10^6^ CFU/mouse), or *S. sonnei* (1 × 10^6^ CFU/mouse) (Table 2). The two-dose pre-exposure of *S. flexneri* 2a provided >90% protection against the homologous *S. flexneri* 2a challenge and the *S. flexneri* 1b challenge, which is in the same serogroup as 2a. In contrast, the protection afforded by *S. flexneri* 2a was only 30% when challenged with *S. sonnei.* Most importantly, 100% of the mice from all groups vaccinated with L-DBF survived and <20% of the mice from the NT-NV group survived (Table 2). Similar results were seen when weight loss was considered (Fig. S4). As expected, mice from T-V groups showed better weight recovery following homologous challenge (2a or 1b) than the NT-V or the T-NV. In contrast, the T-V and NT-V essentially showed equivalent weight gains, which were better than the T-NV group.

**TABLE 2:** Protective efficacy of L-DBF against *Shigella* spp. serotypes following two pre-exposures^*a*^

Set	Grp	Pre-exposure infection	Vaccination	Homologous challenge	Heterologous challenge	
		*S. flexneri* 2a	25 µg L-DBF	*S. flexneri* 2a	*S. flexneri* 1b	*S. sonnei* 53G
A	1	NT	NV	0%	–	–
	2	NT	NV	–	0%	–
	3	NT	NV	–	–	20%
B	4	NT	V	100%	–	–
	5	NT	V	–	100%	–
	6	NT	V	–	–	100%
C	7	T	NV	100%	–	–
	8	T	NV	–	90%	–
	9	T	NV	–	–	30%
D	10	T	V	100%	–	–
	11	T	V	–	100%	–
	12	T	V	–	–	100%

^*a*^ The double pre-exposure groups were treated as follows: Sets A&B were not pre-exposed to *S. flexneri* 2a (NT). Sets C&D were pre-exposed to *S. flexneri* 2a (T). On day 0, sets A&C were administered PBS (NV). Sets B&D were vaccinated with L-DBF (V), V. On day 56, all mice were challenged with shown given as percent survival. –, not applicable.

### *S. flexneri* 2a pre-exposure elicits high levels of bactericidal antibodies that can be blocked by LPS but not by IpaB or IpaD

To determine whether the sera from mouse groups treated once with *S. flexneri* 2a or vaccinated with L-DBF elicited serum bactericidal assay (SBA), we tested the killing activity of the serum immunoglobulins against *S. flexneri* 2a (presented here as killing index or KI). Sera from both T-NV and NT-V mouse groups with one pre-exposure showed SBA, while sera from the NT-NV did not kill bacteria (0%; as baseline) (Fig. S5A). Antibodies from the serum (1:512 dilution) of the T-NV mouse group exhibited >60% bactericidal activity (SBA KI = 1163.11; log = 3.07; *P* < 0.001), while antibodies from the serum of the NT-V mouse group (1:64 dilution) showed an increase in the killing activity around 50% (SBA KI = 59.84; log = 1.78, *P* < 0.001) (Table S1). We propose that the killing activity present in the sera from pre-exposed mouse groups (T) can be attributed to anti-LPS antibodies, while the activity present in the L-DBF vaccinated mice (V) can be attributed to anti-IpaD and anti-IpaB antibodies. To test this theory, we added IpaD, IpaB or LPS to the SBA mixture to block SBA activity (Fig. S5B through D). Indeed, the bactericidal antibodies in the serum from T-NV were blocked by LPS, but not by IpaB or IpaD (*P* < 0.05) when comparing the outcomes of SBA from 2 µg, 1 µg, or 0.5 µg LPS dilution point to those from higher dilution points (Fig. S5B through D). As expected, LPS did not affect the killing activity of the serum from the NT-V group (*P* > 0.999 among all LPS dilution points). It should be noted here that the sera from the NT-V was used at 1:64 since a lower dilution, which had higher SBA activity, would have consumed the available pooled serum quickly, thereby preventing completion of these studies. Nevertheless, while the SBA activity of the 1:64 dilution of NT-V serum was 50%, it remained at 50% regardless of the amount of LPS used in the competition assay. IpaB, but not IpaD, blocked the SBA antibodies in the NT-V mouse group, with *P* < 0.05 when comparing the outcomes of SBA from 2 µg, 1 µg, 0.5 µg, or 0.25 µg IpaB dilution point to those from higher dilution points (Fig. S5C and D). These results suggest that though antibodies in the sera from both T-NV and NT-V mouse groups showed high SBA activity, such activity can be attributed to distinct antigens. Although having a higher SBA KI (log10 ~3), the high SBA antibody levels elicited from a single pre-exposure did not elicit protection against lethal *Shigella* challenges.

**Fig 2:**
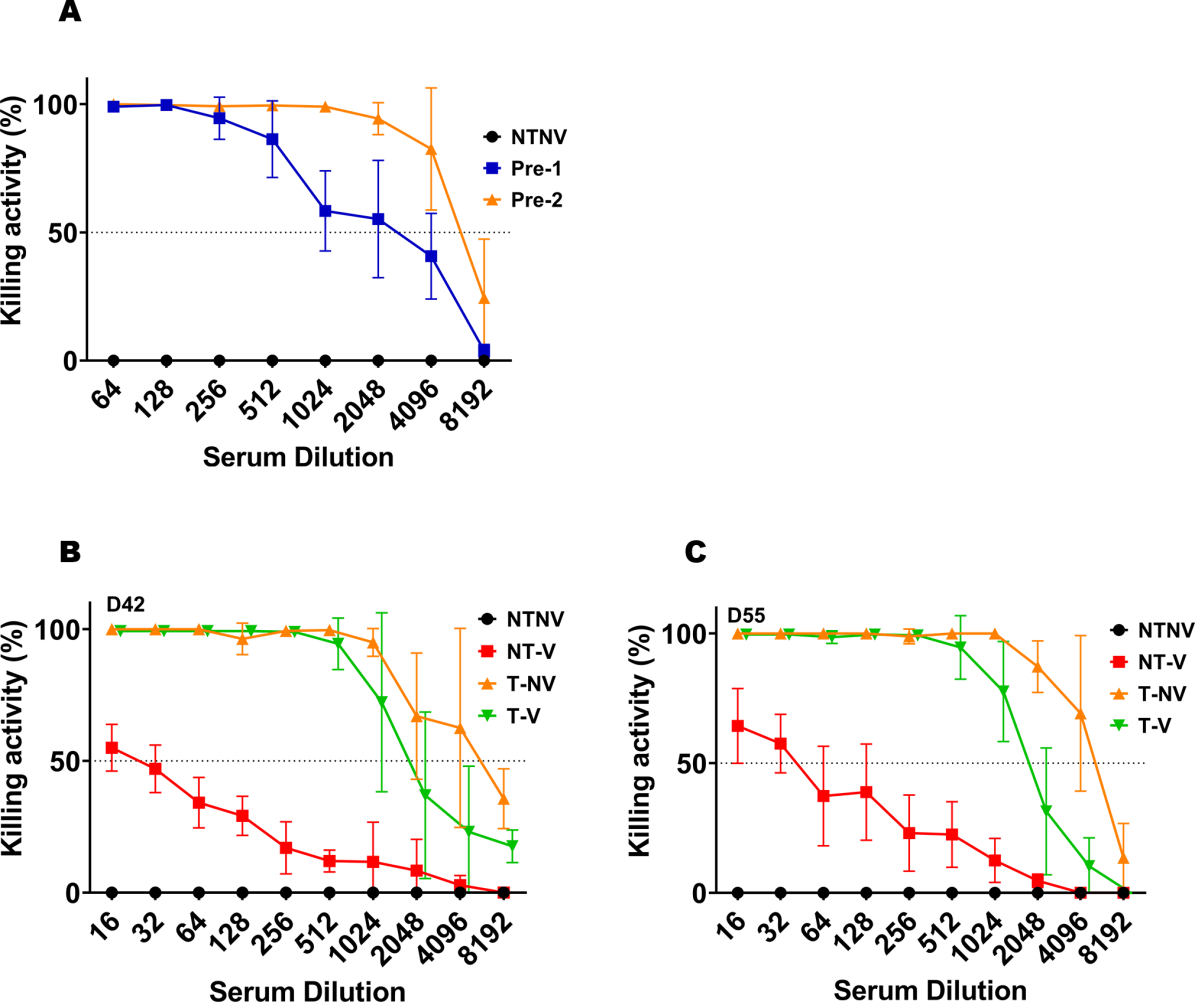
Serum bactericidal assay (SBA) in serum from mouse groups after two *S. flexneri* pre-exposures. The killing activity (%) in serially diluted serum collected on day 28 after the first pre-infection (pre-1) or on day 14 after the second pre-infection (pre-2) is shown (A). The killing activity (%) in serially diluted serum collected on day 42 ( B) or day 55 (C) from NT-V, T-NV, or T-V groups is shown. A dashed horizontal line indicates 50% killing. CFU counts obtained from wells incubating with serum from the NT-NV groups were used as 0% killing (baseline). Killing activity (%) = (spots in NTNV well − spots in test well)/spots in NT-NV well. Duplicate experiments with triplicate wells for each test point are presented. Mann-Whitney test was used for analysis. *P < 0.05; **P < 0.01; ***P < 0.001.

After the first pre-exposure, mouse serum showed a KI of 1,116.68 (log = 3.05), while after the second exposure, the KI was increased to 10,753.53 (log = 4.03) (Fig. 2A). The difference in the SBA between the first and second pre-exposures was statistically observed after a 1:1024 serum dilution (*P* < 0.01) (Table S2). The SBA activity for sera from mice after the first and second exposures were not affected by the addition of IpaB or IpaD but was by purified LPS (*P* < 0.05) when comparing the outcomes of SBA from lower LPS dilution points to those from higher dilution points (Fig. S6). Regardless of group, when the serum collected on day 42 (14 days after the second booster) is compared with that collected on day 55 (27 days after the second booster), SBA activity for the NT-V group was not significantly affected over the course of the L-DBF vaccination period (at 1:4,096 dilution, killing activity dropped from 2.8% to 0%) (Fig. 2B and C). In contrast, the SBA KI was reduced in the T-V group (SBA KI = 2,629.48–2,920.9; log = 3.41–3.46) compared with T-NV (SBA KI = 11858.07–13812.4; log = 4.07–4.14) on day 42 and 55. This is especially true on day 55 since after the 1:1024 serum dilution, the SBA of the serum from T-V group was significantly lower than for T-NV (on day 42, *P* values of T-NV vs T-V were <0.15 at a 1:1,024 serum dilution, and *P* = 0.032 at 1:8,192) (Tables S3 and S4). This change might have been caused by the reduction in the number of LPS targeting bactericidal antibodies. When comparing the 1:8,192 serum dilution between day 42 and 55, killing activity of T-NV dropped from 35% to 13.5% (*P* < 0.001 on day 55 at 1:8,192 compared to NT-NV), while the killing activity of T-V dropped from 18% to 0% (*P* > 0.999 on day 55 at 1:8,192 compared to NT-NV) (Table S4). Although SBA KI, which is based upon a midpoint value, did not show a significant change between day 42 and 55, the drop on killing activity at the end of dilution point suggested that the SBA activity would be reduced once stopping the pre-exposure or vaccination. Thus, bacterial clearance by this mechanism would be significantly less effective.

**Fig 3:**
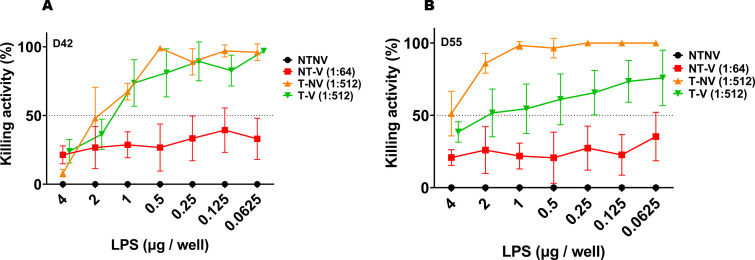
SBA in serum from mouse groups after two *S. flexneri* pre-exposures in competition with exogenously added *S. flexneri* 2a LPS. The killing activity (%) for serum collected on day 42 (A) or day 55 (B) is shown for the NT-V group (1:64 dilution), T-NV group (1:512 dilution) or T-V group (1:512 dilution) in competition with added LPS that is serially diluted. A dashed horizontal line indicates the 50% killing threshold. The viable CFU obtained for wells incubated with serum from the NT-NV groups were used as the 0% killing baseline. Killing % = (spots in NT-NV well − spots in test well)/spots in NT-NV well. Experiments were performed in duplicate with triplicate wells for each test point. The Mann-Whitney test was used for statistical comparisons. *P < 0.05; **P < 0.01; ***P < 0.001.

**Fig 4:**
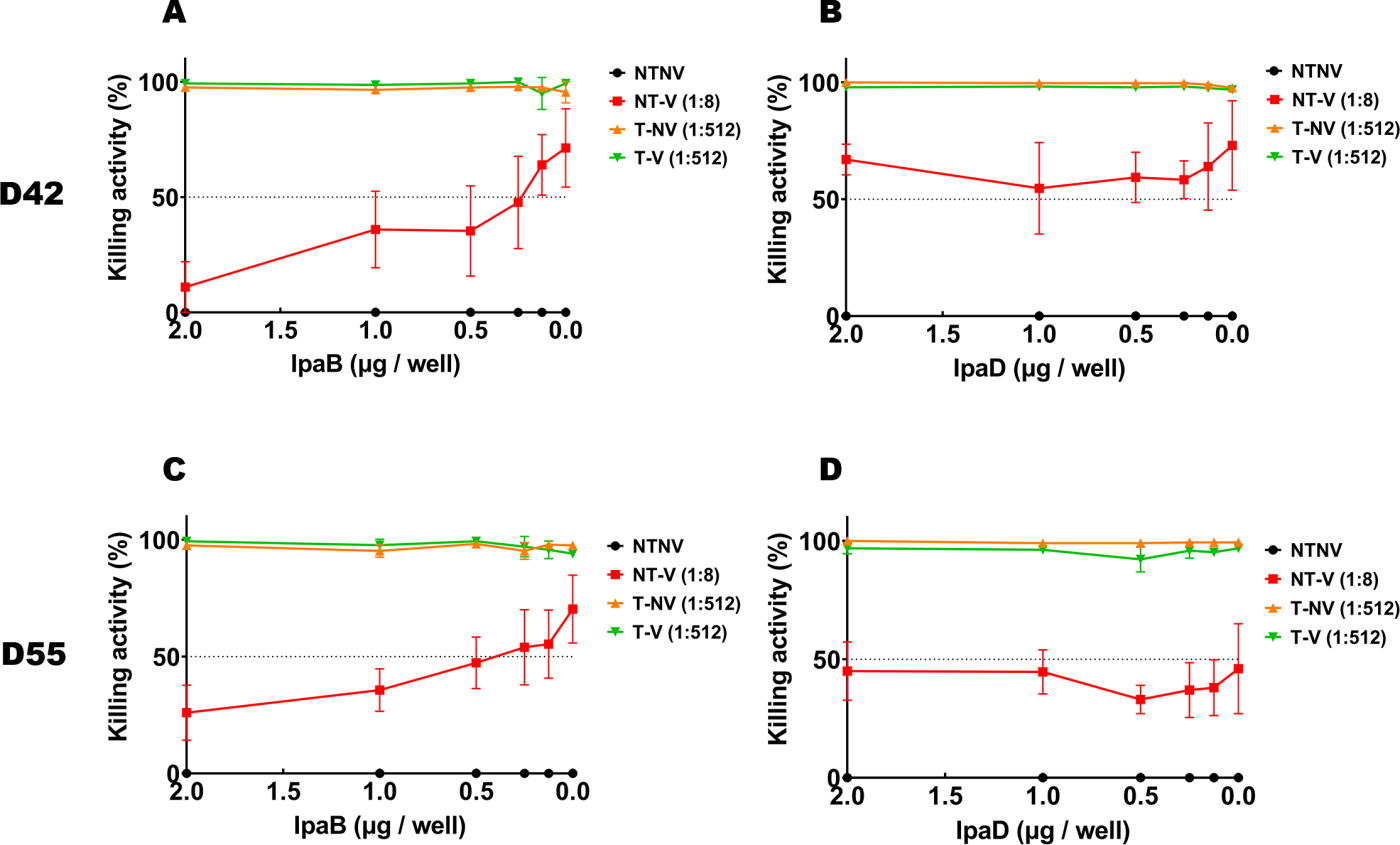
SBA for serum from mouse groups after two *S. flexneri* pre-exposures in competition with exogenously added IpaB or IpaD. The killing activity (%) in serum collected on day 42 (A) or day 55 (C) from NT-V groups (1:8), T-NV groups (1:512) or from T-V groups (1:512) in competition with serially diluted IpaB is shown. The killing activity (%) in serum collected on day 42 (B) or day 55 (D) from NT-V groups (1:8), T-NV groups (1:512) or from T-V groups (1:512) in competition with serially diluted IpaD is shown. The dashed horizontal line indicates the 50% killing threshold. CFU counts obtained with wells incubating with serum from the NT-NV groups were used as the 0% killing baseline. Killing % = (spots in NT-NV well − spots in test well)/spots in NT-NV well. Experiments were performed in duplicate with triplicate wells for each test point. The Mann-Whitney test was used for analysis. *P < 0.05; **P < 0.01; ***P < 0.001.

We next assessed the killing activity of the different pooled sera by using *S. flexneri* 2a LPS as a competitor for the bactericidal function (Fig. 3). SBA activity from NT-V mice was not affected by the addition of LPS with no significant difference seen at the different LPS concentrations. In contrast, the SBA for the T-NV and T-V mice could be reduced by competition with LPS in a dose-dependent manner (*P* < 0.05) as shown by comparing the SBA results using 4 µg, 2 µg, or 1 µg LPS concentrations to the more diluted LPS concentrations. Serum from T-V group only had 75.8% killing ability with 0.625 µg LPS, whereas the SBA from T-NV mice was not affected by the same amount of LPS on day 55 (*P* = 0.002) ( Tables S5 and S6). Meanwhile, the SBA for the NT-V mice mainly targeted the IpaB rather than IpaD since the SBA activity could be readily inhibited in a concentration dependent manner by adding IpaB. The killing activity for NT-V pooled serum in the presence of 2 µg or 1 µg IpaB was significantly lower than that for those without IpaB added on day 42 or 55 (*P* < 0.05). This was not the case for the addition of IpaD (*P* > 0.1 among different IpaD dilution points on day 42 or 55) (Fig. 4; Tables S7 to S10). These results suggest that while SBA in both pre-exposed mouse and L-DBF vaccinated mouse groups showed high bactericidal activity, such activity could be attributed to distinct antigens. With respect to the mice exposed once versus twice with *S. flexneri* 2a, the SBA KI attributable to anti-LPS antibodies suggests that an SBA KI of 10^3^ (seen for the single exposure mice) was not sufficient to protect mice from a *Shigella* lethal challenge. Rather, it appears that an SBA KI of potentially >10^4^ (seen for the double exposure mice) may need to be attained to protect the mice against homologous *Shigella* challenge without the support of vaccination with L-DBF.

### *Shigella* pre-infection tends to elicit IL-6 while vaccination with L-DBF favors induction of IFN-γ and IL-17

**Fig 5:**
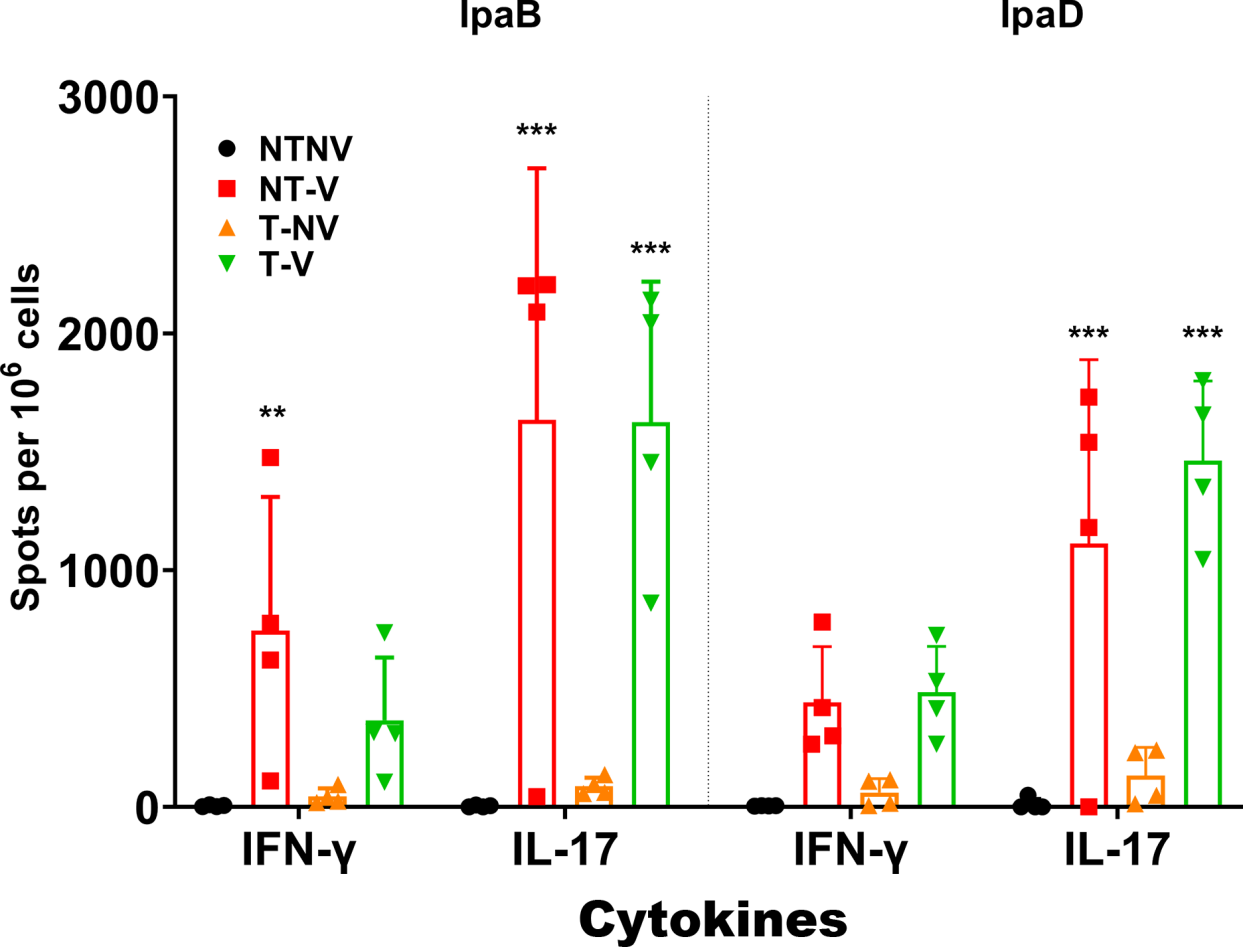
Frequency of IL-17 and IFN-γ secreting cells following antigen-specific stimulation. Lung single cell suspensions were used to assess antigen-specific IFN-γ and IL-17 secreting cells using ELISpot analysis. Cells from each animal group (using the same designations as in the previous figures) were incubated with 10 µg IpaB (left side) or IpaD (right side). IFN-γ and IL-17 secreting cells were then enumerated by ELISpot as described in Materials and Methods and are presented here as spot-forming cells/10^6^ cells. The data are plotted as means ± SD for individual mice in each group. Significance was calculated by comparing groups that were unvaccinated (PBS) and mice vaccinated with antigens using two-way ANOVA. *P < 0.05; **P < 0.01; ***P < 0.001.

To assess why vaccinated mice from NT-V groups have a relatively lower SBA KI (<100) but are still able to be protected from lethal challenge from homologous and heterologous *Shigella* serotypes, we harvested lungs and spleens from all four groups prior to challenge to assess their immune responses. Cell suspensions from each organ were stimulated with IpaB or IpaD and the frequency of IL-17 and IFN-γ secreting cells were enumerated by ELISpot (Fig. 5; Fig. S7). The L-DBF vaccinated mice exhibited comparably higher frequencies of IL-17 secreting cells in lungs when compared to the NT-NV mice, regardless of pre-exposure status. Likewise, frequencies of IFN-γ secreting cells in vaccinated mice were visibly higher when compared to NT-NV mice (IpaD IFN-γ secreting cells of NT-V vs NT-NV: *P* = 0.174; IpaB IFN-γ secreting cells of T-V vs. NT-NV: *P* = 0.306; IpaD IFN-γ secreting cells of T-V vs NTNV: *P* = 0.122). NT-V mice induced higher IFN-γ and IL-17 secreting cells in the spleen while T-V mice did not (Fig. S7). This is despite the fact that all the pre-exposed mice exhibited strong antibody responses against both proteins and IpaB in particular.

**Fig 6:**
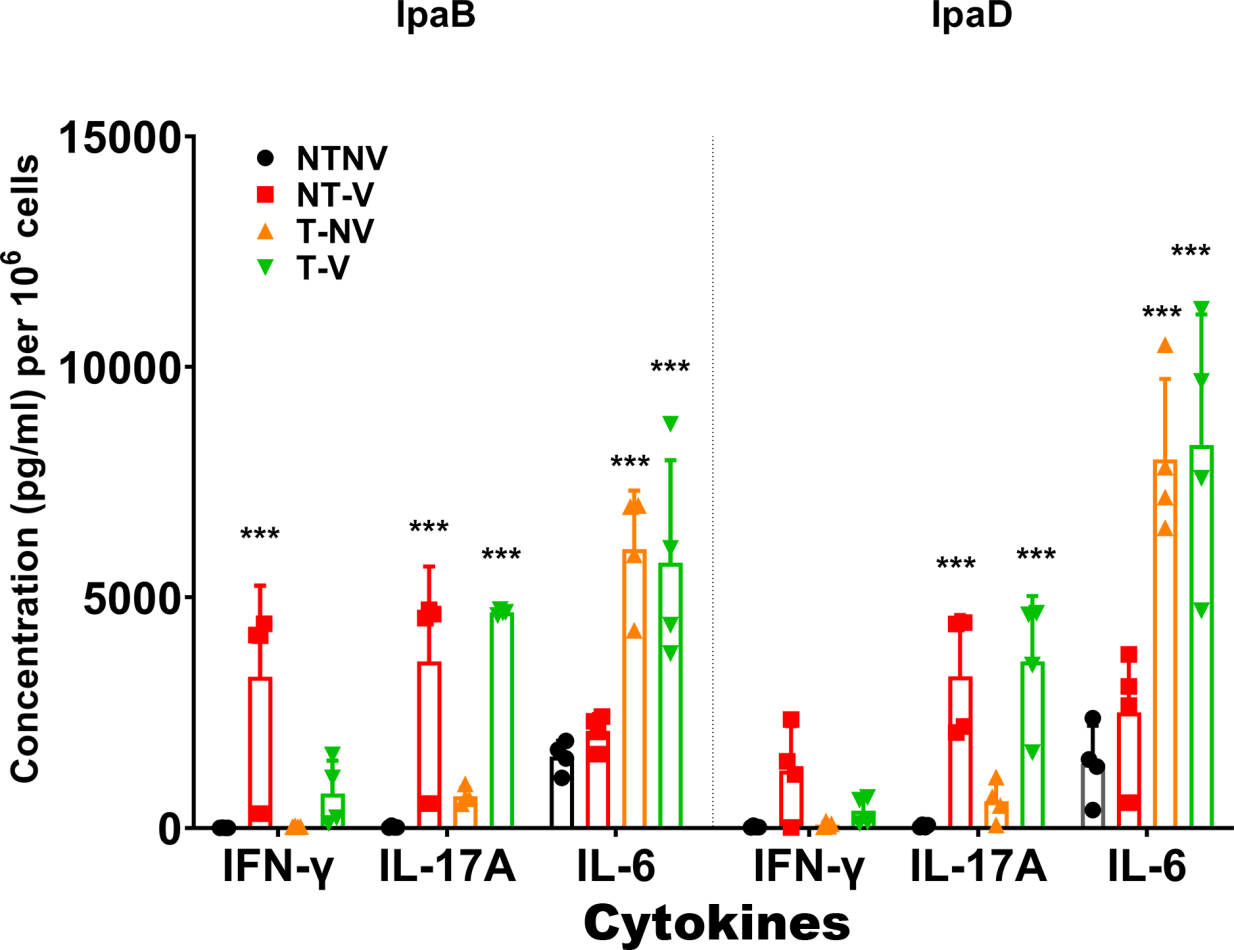
Quantification of T cell-related cytokines secreted from lung cells after stimulation with IpaB or IpaD. The lung single cell suspensions described in Fig. 6 were also used to assess the amount of antigen-specific IFN-γ, IL-17 and IL-6 secreted from these cells. Cells were incubated with 10 µg IpaB (left) or IpaD (right). Cytokine levels were then measured by Meso Scale Discovery analysis as per the manufacturer’s specifications and are presented here as pg/mL/10^6^ cells. The data are plotted as the mean ± SD for the individual mice in each group. Significance was calculated by comparing groups that were unvaccinated (PBS) and mice vaccinated with antigens using two-way ANOVA. *P < 0.05; **P < 0.01; ***P < 0.001.

We quantified overall cytokine levels secreted from these cells following stimulation with IpaB or IpaD (Fig. 6; Fig. S8). Higher levels of IFN-γ and IL-17A were secreted from lung cells from NT-V groups after stimulation with IpaB or IpaD than the NT-NV groups (IpaD IFN-γ of NT-V vs NT-NV: *P* = 0.085). Lung cells from T-V groups secreted significantly higher levels of IL-17A and IL-6 but not IFN-γ (IpaB: *P* = 0.124; IpaD: *P* = 0.105). Pre-exposure mice displayed higher IL-6 relative to the NT groups (Fig. 6). For the spleen cells, only the NT-V mice exhibited higher levels of IFN-γ (IpaB: *P* = 0.067; IpaD: *P* = 0.029), IL-17 (IpaB: *P* = 0.021; IpaD: *P* = 0.022), and IL-6 (IpaB: *P* = 0.022; IpaD: *P* = 0.004). (Fig. S8). These cytokine responses indicate that there are differences in the nature of the immune responses induced in mice by pre-exposure to *Shigella* versus those vaccinated with L-DBF. The IL-17 responses to these proteins by mouse lungs were dependent upon L-DBF vaccination and were not noticeably affected by the pre-existing immune response from pre-exposure. It may be this response that leads to serotype-independent cross-protection against lethal *Shigella* challenge.

## DISCUSSION

Shigellosis is an important public health issue worldwide. *Shigella* infections can have high morbidity and mortality among children under 5 years old, especially those living in developing countries where access to basic life-saving treatments and hygienic resources are limited (23). Although the mortality of shigellosis is on the decline, morbidity remains high and antibiotic resistance is rapidly emerging, which calls for a broad, economic, and effective vaccine against shigellosis. Since most of the target population for a *Shigella* vaccine reside in low-middle income countries, major concerns that must be considered in developing a vaccine are the production costs, the need (or the lack thereof) for a reliable cold chain, and the lack of public health resources. These factors make it difficult for the LMICs to acquire the stockpile needed to secure adequate dosage for their countries. Thus, safe vaccines in simple formulations, offering broad-spectrum protection are required to ensure cost effectiveness, as well as their efficient use.

Our previous studies have shown that L-DBF induces protective immune responses against five unique *Shigella* spp. serotypes with the protective response appearing to correlate with the levels of secreted IL-17 and IFN-γ at the site of infection (19). A drawback of this prior work, however, is the absence of understanding of the immunity against *Shigella* spp. in an endemically infected population. Most LMICs are endemic for shigellosis and a dampened immune response in pre-exposed populations would lessen the impact of an L-DBF subunit vaccine. While we realize that there are certain limitations in mice for this study, we assessed the immune response in mice previously exposed to *S. flexneri* 2a and found that pre-exposure did not dampen the immune response or lessen the efficacy against *Shigella* spp. in L-DBF vaccinated mice. Moreover, we continue to see an interesting interplay between Th17 and Th1 in the form of IL-17A and IFN-γ, respectively, in conjunction with IL-6.

Pre-exposure with one sublethal dose of *S. flexneri* 2a failed to protect mice from subsequent lethal challenge from homologous or heterologous strains. While not protective against subsequent lethal challenge, a single pre-exposure did give rise to antibodies capable of bactericidal activity. The serum bactericidal assay is a complement fixation assay driven by antibodies present in serum that allow for the formation of a membrane attack complex (MAC)(24). While this is an important determinant in the humoral arm of immune response, these findings suggest that there is a strong cellular response needed to neutralize intracellular bacteria like Shigella. Pre-exposure can elicit a strong humoral response, but it appears to fail at generating the appropriate strong T cell response needed for full protection.

In contrast to a single exposure, pre-exposure with two sublethal doses of *S. flexneri* 2a protected the mice from homologous but not heterologous challenge. These results are in line with the original mouse lung model publication where it was demonstrated that two doses of sublethal infection were required to induce 56%–79% protection from a subsequent lethal challenge (22). The literature suggests antibodies are an important determinant for protection against infection by *Shigella* spp. (25), so we examined the generation of bactericidal antibodies in pre-exposed mice. The results revealed a log-fold increase in the SBA titers for LPS in mice subjected to two pre-exposures as compared to mice subjected to a single pre-exposure. Further SBA studies along with ELISAs showed the SBA activity was limited to an increasing anti-LPS response following pre-exposure. This increase suggests a role of B cell epitopes in mounting a protective response against *Shigella* spp., which was found to be limited to homologous challenge only. Although effective, these T-cell independent responses neither generated long-term protection nor did they protect mice from a heterologous challenge. These preliminary results indicate the need for a vaccine with a long-lasting memory response and potentially a protein vaccine that elicits a T-cell dependent response.

Generation of protective immune responses against *Shigella* spp. depends on both B and T cell response, which require the appropriate cytokine response (26). A large spike in lung IL-6 was observed in pre-exposed mice which was absent in the vaccinated mice, while a balanced Th1/Th17 response was not detected in the pre-exposed mice. Since the mouse pre-exposure with a sublethal dose of *S. flexneri* 2a should be representative of a naturally acquired infection, any unique responses in these mice should be considered as an expected outcome of the vaccine in humans in endemic regions. It has been suggested that natural infections “prepare” the immune system to generate a robust antigen-specific, long-lived humoral immune memory, which is true for both viral and non-viral intracellular pathogens (27, 28). Moreover, studies involving *Salmonella enterica* vaccine development showed that mice pre-exposed to *Salmonella* vaccine vectors, compromises their cell-mediated immunity, especially the CD8+ response (29). We did not see this in our study after vaccination with L-DBF despite the presence of high anti-LPS SBA antibodies in the serum of pre-exposed mice. Pre-exposure resulted in significantly higher IL-6 and lower IFN-γ in the lungs of mice, but there were no notable differences seen for TNF-α, IL-12p70, and IL-1β (data not shown). Although important for B cell priming and differentiation to plasma cells and induction of T cell responses, high IL-6 has been shown to be a major factor in derailing the immune system (30 – 32). Previous studies have demonstrated the ability of *Shigella* to impair human T lymphocyte responsiveness (31). It does so by compromising the CD4 T cell F-actin cytoskeleton dynamics resulting in cortical stiffness of the cell. Once infected, the scanning ability of these T cells upon contacting the APCs was found to be impaired, leading to decreased cell-cell contact, compared to the T cells in non-infected humans. Moreover, CD8 T cells fail to respond to other antigens, if presented with *Shigella* (30, 32). The T cell unresponsiveness in *Shigella*-infected cases have been observed by other groups as well, where CD4 T cell motility in the lymph nodes were seen to be dropping drastically (31). The generation of the immune response is also impacted by the length of time between two successive doses and/or infections. Usually, IFN-γ and IL-6 work in tandem, but this was not seen in the present study, where pre-exposure led to a high IL-6 response, but lower IFN-γ secretion in the lung. Interestingly, pre-exposure did not lead to a dampened T cell response during the subsequent vaccination. In our previous studies, we found both Th1 and Th17, along with IL-6, to be important to mount an anti-*Shigella* immunity. All these results suggest that although B cells are primed to generate an immediate short-term anti-*Shigella* immune response, the T cell mediated immune response is dampened following *Shigella* infection. The fact that pre-exposure dampens the immune response and makes it difficult to mount a protective response in already infected mice, suggests that a vaccine seems essential for long term protection.

Since the Th17 response is uninhibited following pre-exposure, L-DBF was able to generate a protective response in these mice. IL-17 is an important cytokine that protects the body from mucosal bacterial challenges. L-DBF vaccinated mice, whether previously exposed or not, generated significantly higher levels of IL-17A. Only the unexposed vaccinated group showed an elevated response of IFN-γ, and all the other groups failed. These results suggest the inability of the pre-exposed mice to generate a strong Th17, Th1 response. It also suggests that these cell types are required for generating a protective immune response against *Shigella* in mice.

In conclusion, L-DBF is a self-adjuvanting subunit vaccine candidate that can induce a protective immune response against multiple *Shigella* serotypes and species, regardless of whether the host has had prior *Shigella* exposure or not. While other subunit vaccines have seen tested (33, 34), the inclusion the two essential and surface exposed immunogens of IpaD and IpaB makes this vaccine platform unique. Then the addition LTA1 and its ability to elicit a strong mucosal immune response suggest that L-DBF is an attractive vaccine candidate for use in regions where shigellosis is endemic.

### Conclusion

Our work provides evidence that L-DBF is a self-adjuvanting vaccine that leads to the development of homologous and heterologous cross-protection against *Shigella* infection. It also appears to elicit an immune response to a highly conserved pair of T3SS proteins with a cytokine profile that is tailored toward clearance of *Shigella* from mucosal sites. Such cross-protective immunogenicity is not disrupted by prior exposure to the target pathogen, thereby suggesting that L-DBF can be used in those areas where shigellosis cases are common.

## MATERIALS AND METHODS

### Materials

Unless otherwise noted, all reagents were from Sigma or Thermo Fisher and were chemical grade or higher. *S. flexneri* 2a 2457T was provided by A. T. Maurelli, University of Florida, Gainesville, FL. *S. flexneri* 1b and *S. sonnei* 53G were provided by Eileen Barry, University of Maryland School of Medicine, Baltimore, MD. *Shigella* LPS was provided by R. K. Ernst (University of Maryland School of Dentistry).

### Protein production

Production of IpaD, IpaB, and L-DBF has been described in detail previously (16, 19). Briefly, plasmids expressing IpaD, IpaB, or L-DBF were used to transform *E. coli* Tuner (DE3) cells. The transformed bacteria were grown in a 10 L bioreactor. Protein expression was induced with 1 mM IPTG and the bacteria grew at 37°C for an additional 3 hours. The bacteria were collected by centrifugation, resuspended in IMAC binding buffer (20 mM Tris-HCl pH 7.9, 500 mM NaCl, and 10 mM imidazole) with 0.1 mM AEBSF, and lysed using a microfluidizer at 18,000 psi with three passes. The cellular debris was removed by centrifugation and the clarified supernatant applied to a nickel-charged IMAC column. The His-tag IpaD (HT-IpaD) was then eluted with IMAC elution buffer (20 mM Tris-HCl pH 8.0, 500 mM NaCl, and 500 mM imidazole). The HT-IpaD was applied to a Q column to remove residual LPS and dialyzed against PBS and stored at −80°C. IpaB and L-DBF were expressed with HT-IpgC, the cognate IpaB chaperone. The IpaB/HT IpgC or L-DBF/HT-IpgC was purified using the IMAC procedure as for HT-IpaD. The complex was then dialyzed, loaded onto a Q column, and eluted with increasing NaCl. Lauryldimethylamine oxide (LDAO) was then added to the pooled fractions at a final concentration of 0.1% to release the HT-IpgC. The LDAO-treated IpaB/HT-IpgC or L-DBF/HT-IpgC complexes were then passed over a second IMAC column with the IpaB and L-DBF present in the flow-through. These were then dialyzed into PBS with 0.05% LDAO and stored at −80°C. LPS levels were determined using a NexGen PTS system with EndoSafe cartridges (Charles River Laboratories, Wilmington, MA). All proteins had LPS levels <5 EU/mg protein. Protein yields are typically 1 mg L-DBF per liter of culture, however, optimization of induction and production are still under way.

### Mouse *S. flexneri* 2a pre-exposure, L-DBF vaccination, and challenge

*S. flexneri* 2a, *S. flexneri* 1b, or *S. sonnei* were cultivated on tryptic soy agar (TSA) plates containing 0.025% Congo red overnight. They were grown in tryptic soy broth (TSB) at 37°C, at 200 rpm until the absorbance (A_600_) reached 1. Bacteria were harvested by centrifugation and resuspended in PBS.

For the single pre-exposure study, female 6- to 8-week-old BALB/c mice (*n* = 10/group) were exposed to PBS or 6 × 10^4^ CFU/30 µL *S. flexneri* 2a on day −60 (60 days prior to the first vaccination). For the two-dose pre-exposure study (Table 1), female 6- to 8-week-old BALB/c mice (*n* = 14/group; 10 for challenge and 4 for pre-challenge immune response assessment) were used. The no treatment groups (sets A and B) were exposed to PBS on days −56 and −28. The treatment groups (sets C and D) were exposed to 1 × 10^5^ CFU/30 µL of *S. flexneri* 2a on day −56 and 6 × 10^5^ CFU/30 µL of *S. flexneri* 2a on day −28. All mice were weighed once daily, and the health score monitored twice daily for 14 days after each pre-exposure. After recovering until day 0, three groups of the NT mice (set A) and three groups of the T mice (set C) were vaccinated with 25 µg L-DBF in 30 µL per mouse on days 0, 14, and 28 while the other six groups were vaccinated with PBS (NV) (sets B and D). On day 56, one group (*n* = 10) from each of the four sets were challenged with *S. flexneri* 2a, *S. flexneri* 1b, or *S. sonnei* (see Table 1). Mice were monitored twice daily for weight loss and health scores for 2 weeks. Mice were euthanized if their weight loss exceeded 25% of their original weight for more than 72 hours or their blood glucose reached ≤100 mg/dL if they received a poor health score. The mouse animal protocols were reviewed and approved by the University of Kansas Institutional Animal Care and Use Committee Practices (protocol AUS 222–01).

### IgG and IgA ELISAs

For the one dose pre-exposure mice, blood and fecal samples were collected on days 0, 13, 27, 41, and 55 for determination of immunoglobulin titers as described previously with minor modifications (22). For the two dose pre-exposure mice, samples were collected at days −56,–28, −14, 0, 14, 28, 42, and 55. To determine anti-IpaD or anti-IpaB titers, wells were coated with 100 ng IpaB or IpaD in 100 µL PBS and incubated at 37°C for 3 hours. Wells were then blocked with 10% nonfat dried milk in PBS overnight. Sera were added to the wells in duplicates as the source of primary antibody and incubated for 2 hours at 37°C. After washing with PBS containing 0.05% Tween 20, HRP-conjugated secondary antibodies [IgG(H+L), 1:1,000; IgA, 1:500; IgM, 1:1,000] were added and incubated for 1 hour at 37°C. After an additional wash, OPD substrate (o-phenylenediamine dihydrochloride) was added and detected at 490 nm using an ELISA plate reader. Endpoint titers were determined by fitting antibody titrations to a five-parameter logistic model.

For determining the anti-LPS titers, wells were coated with 0.5 µg of *S. flexneri* 2a LPS in 100 µL 0.05 M carbonate buffer (pH 9.6) for 1 hour at 37°C. After washing, wells were blocked with 10% nonfat dried milk in PBS for 1 hour at 37°C. After removing the milk and washing, sera were added to the wells in duplicates as primary antibody for overnight incubation at room temperature. The wells then were washed, the HRP-conjugated secondary antibody [IgG(H+L), 1:1,000; IgA, 1:500; IgM, 1:1,000] was added and the plate was incubated overnight at room temperature. After an additional wash, OPD substrate was added and the plates were read at 490 nm using an ELISA plate reader. Endpoint titers were determined by fitting antibody titrations to a five-parameter logistic model.

### Serum bactericidal assay

The serum bactericidal assay (SBA) was modified by using high-throughput imaging of the bacteria on filter plates as previously described (35). Briefly, heat-inactivated serum, produced by pooling sera from each mouse of the group, was diluted twofold with PBS in triplicate. A portion (90 µL) of the diluted serum and baby rabbit complement (Cedarlane, Burlington, NC) were added to each well of a 96-round well round bottom plate. A single colony of *S. flexneri* 2a grown on Congo Red TSA plate was sub-cultured in 10 mL of TSB at 37°C with shaking at 200 rpm and grown until the A_600_ reached 0.2. *S. flexneri* (at 1 × 10^4^ CFU/10 µL) was added to each well and the plates placed at 37°C with 200 rpm shaking for 1 hour. A portion (20 µL) of each mix condition was transferred to ethanol-wetted wells of Millipore multiscreen HV filtration plates and the liquid removed by vacuum. Each plate was placed into a Ziploc bag and incubated at 37°C and 5% CO_2_ overnight. The next morning, Coomassie blue R-250 (100 µL of 0.01% solution) was added to each well and quickly removed by vacuum. A methanol-acetic acid destain solution (100 µL) was then added to each well with shaking at room temperature for 10 minutes. The destain solution was removed by vacuum and the plastic bottom of the filter plate was removed and allowed to air dry before counting. The CFUs were enumerated by a CTL (Cellular Technology Limited) immunospot reader.

For the competitive SBA, 4 µg of LPS of *S. flexneri* 2a 2,457T, 2 µg of IpaB, or IpaD in 45 µL PBS was added to the wells following twofold dilutions done in triplicate. A portion (90 µL) of a pooled mixture of serum from pre-infected mice (1:512) or from L-DBF immunized mice (1:8 for the IpaB or IpaD; 1:64 for the LPS) was combined with baby rabbit complement in the appropriate wells. After gentle shaking at room temperature for 30 minutes, *S. flexneri* (1 × 10^4^ CFU/10 µL) was added to each well. The remaining steps were performed as above. The killing activity was measured by the following formula: killing % = (spots in NTNV well – spots in test well)/spots in NTNV well. The number of spots in NTNV wells were statistically insignificantly different than the wells containing the complement and the bacteria. Thus, NTNV wells were considered as a baseline and a negative control group. The SBA KI was calculated by 10^{log*X*1 + [(*Y*50 − Y1) × (log*X*2 − log*X*1)]/(*Y*2 − *Y*1)}^.

### IFN-γ or IL-17A ELISpot assays

Mouse cells were isolated from spleens and lungs as previously described (19). The cells were incubated for 24 hours at 37°C in the presence of 5 µg/mL IpaB or IpaD in plates coated with antibodies against IFN-γ or IL-17 using a FluoroSpot assay as per manufacturer’s specifications (CTL). The cytokine secreting cells were quantified using a CTL Immunospot reader.

### Cytokine determinations

Splenocytes and lung cells were incubated with 10 µg/mL IpaB, IpaD, or PBS for 48 hours at 37°C. Supernatants were collected and analyzed with U-PLEX kits for cytokines according to manufacturer’s specifications. Cytokine concentrations were determined using an MSD plate reader with associated analytical software (Meso Scale Discovery, Rockville, MD). While multiple cytokines were measured, the three that were focused on in this study are IL-17A, IFN-γ and IL-6 because the others that were tested did not show significant changes.

### Statistics

GraphPad Prism 9.0.1 and Python were used for graphs and statistical analysis. ANOVA test was used for cytokine analysis. Log-rank (Mantel-Cox) tests were used for survival tests. Mann-Whitney tests were used for SBA analysis. *P < 0.05; **P < 0.01; ***P < 0.001.

## SUPPLEMENTAL MATERIAL

Supplemental material spectrum.00062-23-s0001.pdf Fig. S1 to S8; Tables S1 to S10.

ASM does not own the copyrights to Supplemental Material that may be linked to, or accessed through, an article. The authors have granted ASM a non-exclusive, world-wide license to publish the Supplemental Material files. Please contact the corresponding author directly for reuse.

## References

[1] Kotloff KL , Riddle MS , Platts-Mills JA , Pavlinac P , Zaidi AKM . 2018. Shigellosis. Lancet 391:801–812. doi:10.1016/S0140-6736(17)33296-8 29254859

[2] Kolling G , Wu M , Guerrant RL . 2012. Enteric pathogens through life stages. Front Cell Infect Microbiol 2:114. doi:10.3389/fcimb.2012.00114 22937528 PMC3427492

[3] Tallant A , Porter CK , Putnam SD , Tribble DR , Hooper TI , Riddle MS . 2014. Relative cost-effectiveness of a norovirus vaccine in the deployed military setting compared to a vaccine against Campylobacter sp., ETEC, and Shigella sp. Vaccine 32:5156–5162. doi:10.1016/j.vaccine.2014.07.070 25086264

[4] Bengtsson RJ , Simpkin AJ , Pulford CV , Low R , Rasko DA , Rigden DJ , Hall N , Barry EM , Tennant SM , Baker KS . 2022. Pathogenomic analyses of Shigella isolates inform factors limiting shigellosis prevention and control across Lmics. Nat Microbiol 7:251–261. doi:10.1038/s41564-021-01054-z 35102306 PMC8813619

[5] Khalil IA , Troeger C , Blacker BF , Rao PC , Brown A , Atherly DE , Brewer TG , Engmann CM , Houpt ER , Kang G , Kotloff KL , Levine MM , Luby SP , MacLennan CA , Pan WK , Pavlinac PB , Platts-Mills JA , Qadri F , Riddle MS , Ryan ET , Shoultz DA , Steele AD , Walson JL , Sanders JW , Mokdad AH , Murray CJL , Hay SI , Reiner RC . 2018. Morbidity and mortality due to Shigella and enterotoxigenic Escherichia coli diarrhoea: the global burden of disease study 1990-2016. Lancet Infect Dis 18:1229–1240. doi:10.1016/S1473-3099(18)30475-4 30266330 PMC6202441

[6] Allen H , Mitchell HD , Simms I , Baker KS , Foster K , Hughes G , Dallman TJ , Jenkins C . 2021. Evidence for re-infection and persistent carriage of Shigella species in adult males reporting domestically acquired infection in England. Clin Microbiol Infect 27:126. doi:10.1016/j.cmi.2020.03.036 32247893

[7] Rew V , Mook P , Trienekens S , Baker KS , Dallman TJ , Jenkins C , Crook PD , Thomson NR . 2018. Whole-genome sequencing revealed concurrent outbreaks of shigellosis in the English orthodox Jewish community caused by multiple importations of Shigella sonnei from Israel. Microb Genom 4:e000170. doi:10.1099/mgen.0.000170 29583113 PMC5885021

[8] Baker KS , Dallman TJ , Field N , Childs T , Mitchell H , Day M , Weill F-X , Lefèvre S , Tourdjman M , Hughes G , Jenkins C , Thomson N . 2018. Genomic epidemiology of Shigella in the United Kingdom shows transmission of pathogen sublineages and determinants of antimicrobial resistance. Sci Rep 8:7389. doi:10.1038/s41598-018-25764-3 29743642 PMC5943296

[9] Baker KS , Dallman TJ , Field N , Childs T , Mitchell H , Day M , Weill F-X , Lefèvre S , Tourdjman M , Hughes G , Jenkins C , Thomson N . 2018. Horizontal antimicrobial resistance transfer drives epidemics of multiple Shigella species. Nat Commun 9:1462. doi:10.1038/s41467-018-03949-8 29654279 PMC5899146

[10] Hawkey J , Paranagama K , Baker KS , Bengtsson RJ , Weill F-X , Thomson NR , Baker S , Cerdeira L , Iqbal Z , Hunt M , Ingle DJ , Dallman TJ , Jenkins C , Williamson DA , Holt KE . 2021. Global population structure and genotyping framework for genomic surveillance of the major dysentery pathogen, Shigella sonnei. Nat Commun 12:2684. doi:10.1038/s41467-021-22700-4 33976138 PMC8113504

[11] Connor TR , Barker CR , Baker KS , Weill F-X , Talukder KA , Smith AM , Baker S , Gouali M , Pham Thanh D , Jahan Azmi I , Dias da Silveira W , Semmler T , Wieler LH , Jenkins C , Cravioto A , Faruque SM , Parkhill J , Wook Kim D , Keddy KH , Thomson NR . 2015. Species-wide whole genome sequencing reveals historical global spread and recent local persistence in Shigella flexneri. Elife 4:e07335. doi:10.7554/eLife.07335 26238191 PMC4522646

[12] Barry EM , Pasetti MF , Sztein MB , Fasano A , Kotloff KL , Levine MM . 2013. Progress and pitfalls in Shigella vaccine research. Nat Rev Gastroenterol Hepatol 10:245–255. doi:10.1038/nrgastro.2013.12 23419287 PMC3747556

[13] Muthuramalingam M , Whittier SK , Picking WL , Picking WD . 2021. The Shigella type III secretion system: an overview from top to bottom. Microorganisms 9:451. doi:10.3390/microorganisms9020451 33671545 PMC7926512

[14] Espina M , Olive AJ , Kenjale R , Moore DS , Ausar SF , Kaminski RW , Oaks EV , Middaugh CR , Picking WD , Picking WL . 2006. IpaD localizes to the tip of the type III secretion system needle of Shigella flexneri. Infect Immun 74:4391–4400. doi:10.1128/IAI.00440-06 16861624 PMC1539624

[15] Olive AJ , Kenjale R , Espina M , Moore DS , Picking WL , Picking WD . 2007. Bile salts stimulate recruitment of IpaB to the Shigella flexneri surface, where it colocalizes with IpaD at the tip of the type III secretion needle. Infect Immun 75:2626–2629. doi:10.1128/IAI.01599-06 17296762 PMC1865747

[16] Martinez-Becerra F.J , Kissmann JM , Diaz-McNair J , Choudhari SP , Quick AM , Mellado-Sanchez G , Clements JD , Pasetti MF , Picking WL . 2012. Broadly protective Shigella vaccine based on type III secretion apparatus proteins. Infect Immun 80:1222–1231. doi:10.1128/IAI.06174-11 22202122 PMC3294653

[17] Martinez-Becerra Francisco J , Chen X , Dickenson NE , Choudhari SP , Harrison K , Clements JD , Picking WD , Van De Verg LL , Walker RI , Picking WL . 2013. Characterization of a novel fusion protein from IpaB and IpaD of Shigella spp. and its potential as a pan-Shigella vaccine. Infect Immun 81:4470–4477. doi:10.1128/IAI.00859-13 24060976 PMC3837967

[18] Norton EB , Lawson LB , Mahdi Z , Freytag LC , Clements JD . 2012. The A subunit of Escherichia coli heat-labile enterotoxin functions as a mucosal adjuvant and promotes IgG2A, IgA, and Th17 responses to vaccine antigens. Infect Immun 80:2426–2435. doi:10.1128/IAI.00181-12 22526674 PMC3416479

[19] Lu T , Das S , Howlader DR , Zheng Q , Ratnakaram SSK , Whittier SK , Picking WD , Picking WL . 2021. L-DBF elicits cross protection against different serotypes of Shigella spp. Front Trop Dis 2. doi:10.3389/fitd.2021.729731

[20] Sellge G , Magalhaes JG , Konradt C , Fritz JH , Salgado-Pabon W , Eberl G , Bandeira A , Di Santo JP , Sansonetti PJ , Phalipon A . 2010. Th17 cells are the dominant T cell subtype primed by Shigella flexneri mediating protective immunity. J Immunol 184:2076–2085. doi:10.4049/jimmunol.0900978 20089698

[21] Livio S , Strockbine NA , Panchalingam S , Tennant SM , Barry EM , Marohn ME , Antonio M , Hossain A , Mandomando I , Ochieng JB , Oundo JO , Qureshi S , Ramamurthy T , Tamboura B , Adegbola RA , Hossain MJ , Saha D , Sen S , Faruque ASG , Alonso PL , Breiman RF , Zaidi AKM , Sur D , Sow SO , Berkeley LY , O’Reilly CE , Mintz ED , Biswas K , Cohen D , Farag TH , Nasrin D , Wu Y , Blackwelder WC , Kotloff KL , Nataro JP , Levine MM . 2014. Shigella isolates from the global enteric multicenter study inform vaccine development. Clin Infect Dis 59:933–941. doi:10.1093/cid/ciu468 24958238 PMC4166982

[22] van de Verg LL , Mallett CP , Collins HH , Larsen T , Hammack C , Hale TL . 1995. Antibody and cytokine responses in a mouse pulmonary model of Shigella flexneri serotype 2a infection. Infect Immun 63:1947–1954. doi:10.1128/iai.63.5.1947-1954.1995 7729907 PMC173248

[23] Hosangadi D , Smith PG , Giersing BK . 2019. Considerations for using ETEC and Shigella disease burden estimates to guide vaccine development strategy. Vaccine 37:7372–7380. doi:10.1016/j.vaccine.2017.09.083 29031690 PMC6892262

[24] Xie CB , Jane-Wit D , Pober JS . 2020. Complement membrane attack complex: new roles, mechanisms of action, and therapeutic targets. Am J Pathol 190:1138–1150. doi:10.1016/j.ajpath.2020.02.006 32194049 PMC7280757

[25] Ndungo E , Pasetti MF . 2020. Functional antibodies as immunological endpoints to evaluate protective immunity against Shigella. Hum Vaccin Immunother 16:197–205. doi:10.1080/21645515.2019.1640427 31287754 PMC7670857

[26] Salgado-Pabón W , Konradt C , Sansonetti PJ , Phalipon A . 2014. New insights into the crosstalk between Shigella and T lymphocytes. Trends Microbiol 22:192–198. doi:10.1016/j.tim.2014.02.002 24613405

[27] Maglione PJ , Chan J . 2009. How B cells shape the immune response against Mycobacterium tuberculosis. Eur J Immunol 39:676–686. doi:10.1002/eji.200839148 19283721 PMC2760469

[28] Turner JS , Kim W , Kalaidina E , Goss CW , Rauseo AM , Schmitz AJ , Hansen L , Haile A , Klebert MK , Pusic I , O’Halloran JA , Presti RM , Ellebedy AH . 2021. SARS-CoV-2 infection induces long-lived bone marrow plasma cells in humans. Nature 595:421–425. doi:10.1038/s41586-021-03647-4 34030176

[29] Sevil Domènech VE , Panthel K , Meinel KM , Winter SE , Rüssmann H . 2007. Pre-existing anti-salmonella vector immunity prevents the development of protective antigen-specific CD8 T-cell frequencies against murine listeriosis. Microbes Infect 9:1447–1453. doi:10.1016/j.micinf.2007.07.010 17913544

[30] Samassa F , Ferrari ML , Husson J , Mikhailova A , Porat Z , Sidaner F , Brunner K , Teo T-H , Frigimelica E , Tinevez J-Y , Sansonetti PJ , Thoulouze M-I , Phalipon A . 2020. Shigella impairs human T lymphocyte responsiveness by hijacking actin cytoskeleton dynamics and T cell receptor vesicular trafficking. Cell Microbiol 22:e13166. doi:10.1111/cmi.13166 31957253 PMC7187243

[31] Salgado-Pabón W , Celli S , Arena ET , Nothelfer K , Roux P , Sellge G , Frigimelica E , Bousso P , Sansonetti PJ , Phalipon A . 2013. Shigella impairs T lymphocyte dynamics in vivo. Proc Natl Acad Sci U S A 110:4458–4463. doi:10.1073/pnas.1300981110 23417297 PMC3606969

[32] Jehl SP , Doling AM , Giddings KS , Phalipon A , Sansonetti PJ , Goldberg MB , Starnbach MN . 2011. Antigen-specific CD8(+) T cells fail to respond to Shigella flexneri. Infect Immun 79:2021–2030. doi:10.1128/IAI.00939-10 21357720 PMC3088127

[33] Pore D , Chakrabarti MK . 2013. Outer membrane protein A (OmpA) from Shigella flexneri 2a: a promising subunit vaccine candidate. Vaccine 31:3644–3650. doi:10.1016/j.vaccine.2013.05.100 23764536

[34] Anam K , Endharti AT , Poeranto S , Sujuti H , Hidayati DYN , Prawiro SR . 2022. Shigella flexneri vaccine development: oral administration of peptides derived from the 49.8 kDa pili protein subunit activates the intestinal immune response in mice. Vet World 15:281–287. doi:10.14202/vetworld.2022.281-287 35400957 PMC8980390

[35] Liu X , Wang S , Sendi L , Caulfield MJ . 2004. High-throughput imaging of bacterial colonies grown on filter plates with application to serum bactericidal assays. J Immunol Methods 292:187–193. doi:10.1016/j.jim.2004.06.021 15350523

