# Supplementary material for: The L-DBF vaccine cross protects mice against different Shigella serotypes after prior exposure to the pathogen
Source: Microbiol Spectr. 2023 Oct 3;11(6):e00062-23. doi: 10.1128/spectrum.00062-23 (PMC10714971; doi:10.1128/spectrum.00062-23)
Supplement: Supplemental material — Fig. S1 to S8; Tables S1 to S10. [file spectrum.00062-23-s0001.pdf]

SUPPLEMENTARY MATERIALS for:

LDBF cross protects mice against different *Shigella* serotypes after early exposure to pathogen.

Ti Lu[1#], Debaki R Howlader[1#], Sayan Das[1§], Zackary K Dietz[1#], Aaron C. Nagel[2], Sean K. Whittier[1#], William D Picking[1¥], and Wendy L Picking[1¥*]

[1]Department of Pharmaceutical Chemistry, University of Kansas, Lawrence, Kansas 66047 and [2]Hafion, Inc, Lawrence, KS 66047

[#]Current address: Bond Life Sciences Center, University of Missouri, Columbia, MO 65211, USA

[§]Current address: University of Maryland-Baltimore School of Dentistry, Microbial Pathogenesis, Baltimore, Maryland, USA.

[¥]Current address: Bond Life Sciences Center and Department of Veterinary Pathobiology, University of Missouri, Columbia, MO 65211, USA.

*Corresponding Author: Wendy L Picking, wendy.picking@missouri.edu

Keywords: Shigellosis; Pre-exposure; IpaB; IpaD; Vaccine; IL-17; IFN-γ; Il-6

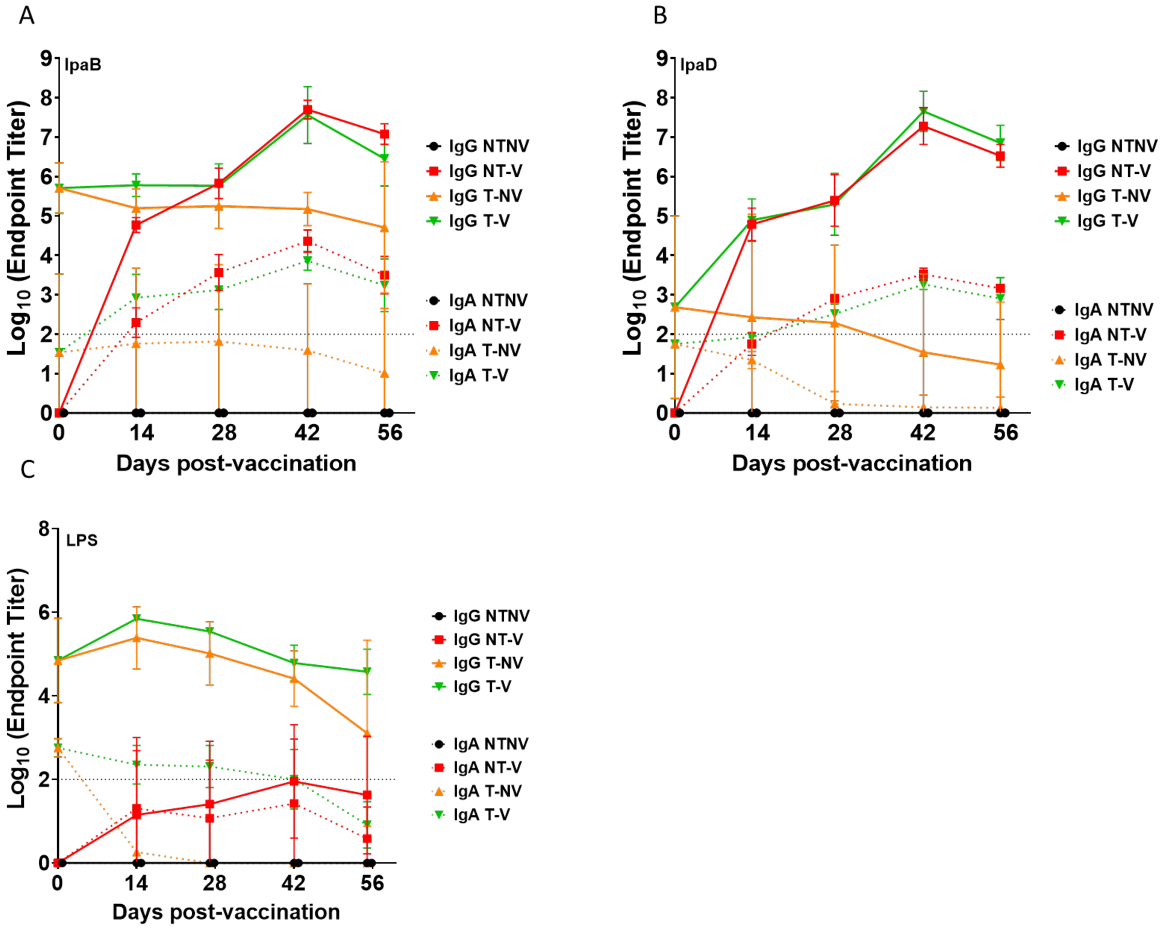

Supplemental Figure 1. Kinetics of serum IgG and fecal IgA titers from mice after single pre-exposure. Mice were vaccinated intranasally (IN) three times (Day 0, 14, and 28). Blood and fecal samples were collected. Kinetics of resulting titers were measured for anti-IpaB (**panel A**), anti-IpaD (**panel B**) or anti-LPS (**panel C**) IgG (**solid**) and IgA (**dotted**) by ELISA. The individual titers are represented as EU ml^{-1}. Each point represents the mean of each group (n=10/group). NT-NV: no pre-exposure and PBS vaccination; NT-V: no pre-exposure and L-DBF vaccination; T-NV: pre-exposure and vaccinated with PBS; T-V: pre-exposure and L-DBF vaccinated. The log = 2 (black dotted line) is the cutting level of the background.

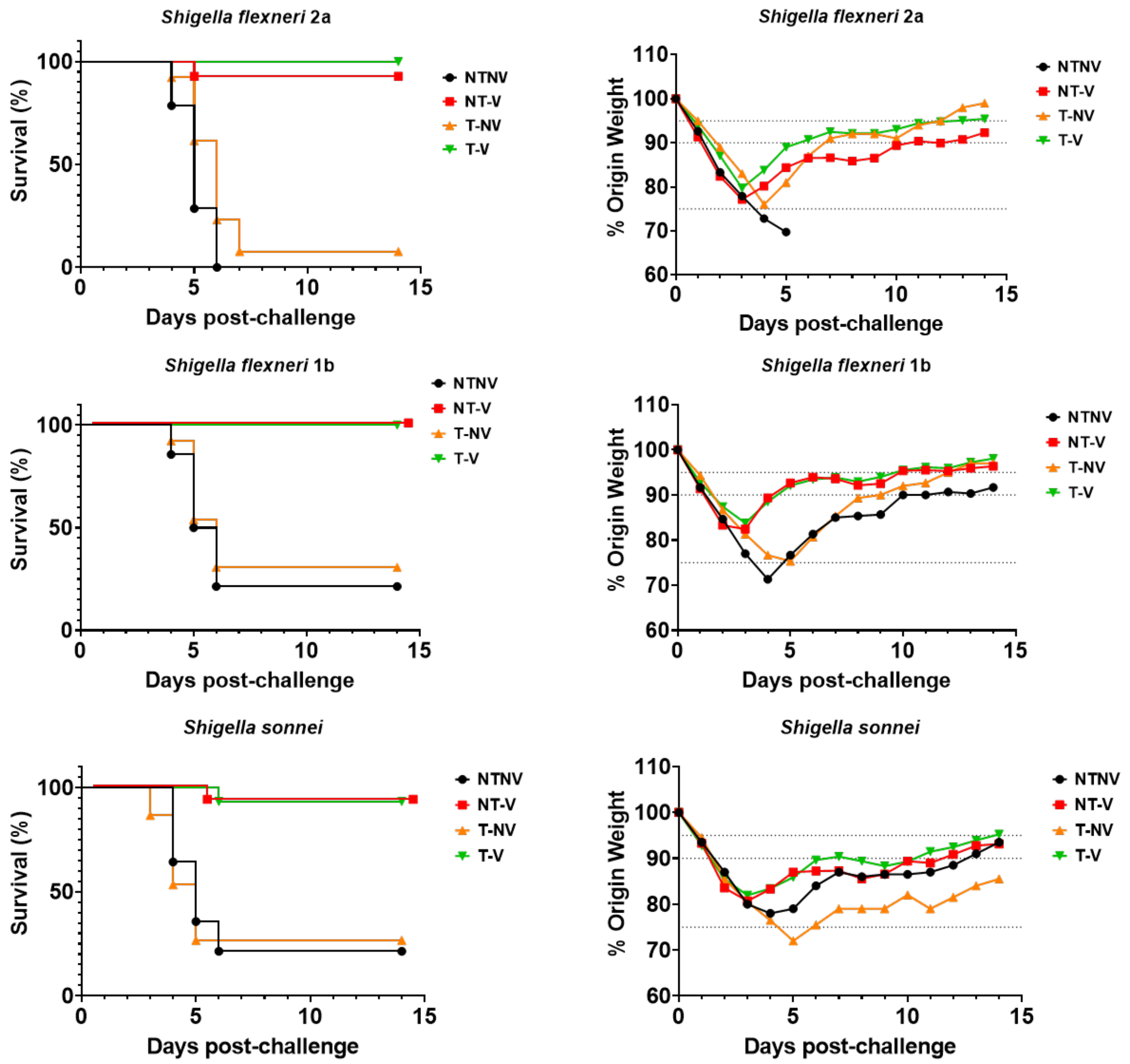

Supplemental Figure 2. Protective efficacy of L-DBF against a *Shigella* lethal challenge in mice. Balb/C mice (n=10) were pre-exposed to a single sublethal dose of *S. flexneri* 2a on day -60 and then vaccinated IN on days 0, 14 and 28 with 25 µg L-DBF. On day 56, the mice were challenged IN with *S. flexneri* 2a, *S. flexneri* 1b or *S. sonnei*.

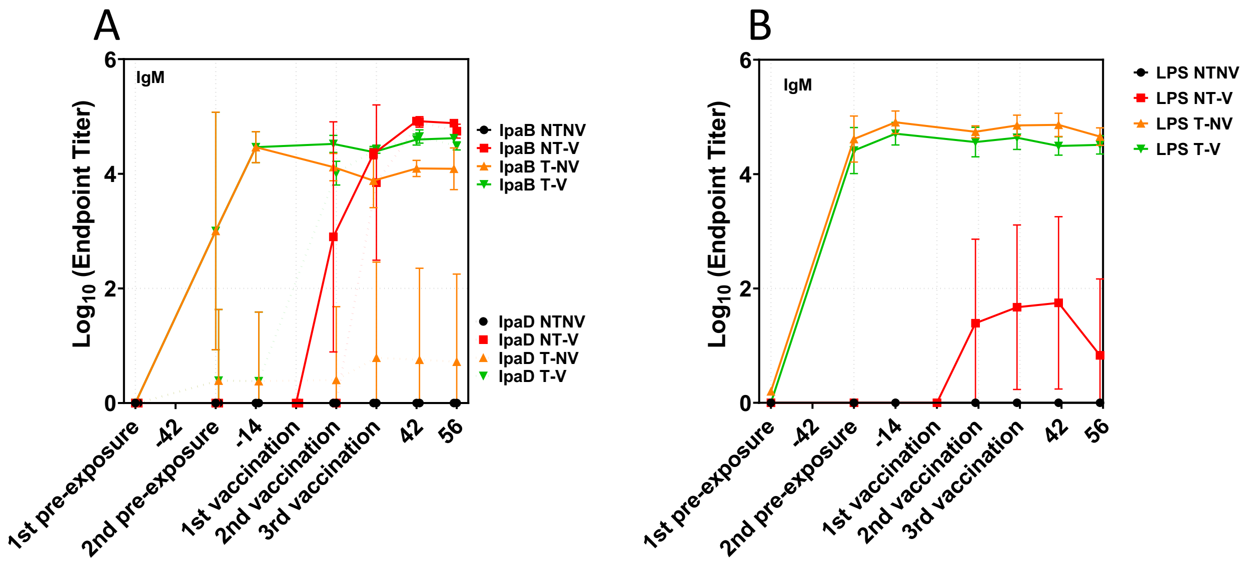

Supplemental Figure 3. **Kinetics of serum IgM responses from mice with two sublethal doses of *S. flexneri* 2a on days -56 and -28**. Mice were then vaccinated IN three times (days 0, 14, and 28). Blood and fecal samples were collected, and the titers determined for anti-IpaB (**solid**) or IpaD (**dotted**) (**Panel A**) or anti-LPS (**Panel B**) IgM by ELISA. The individual titers are represented as EU ml^{-1}. Each point represents the mean of each group (n=10/group).

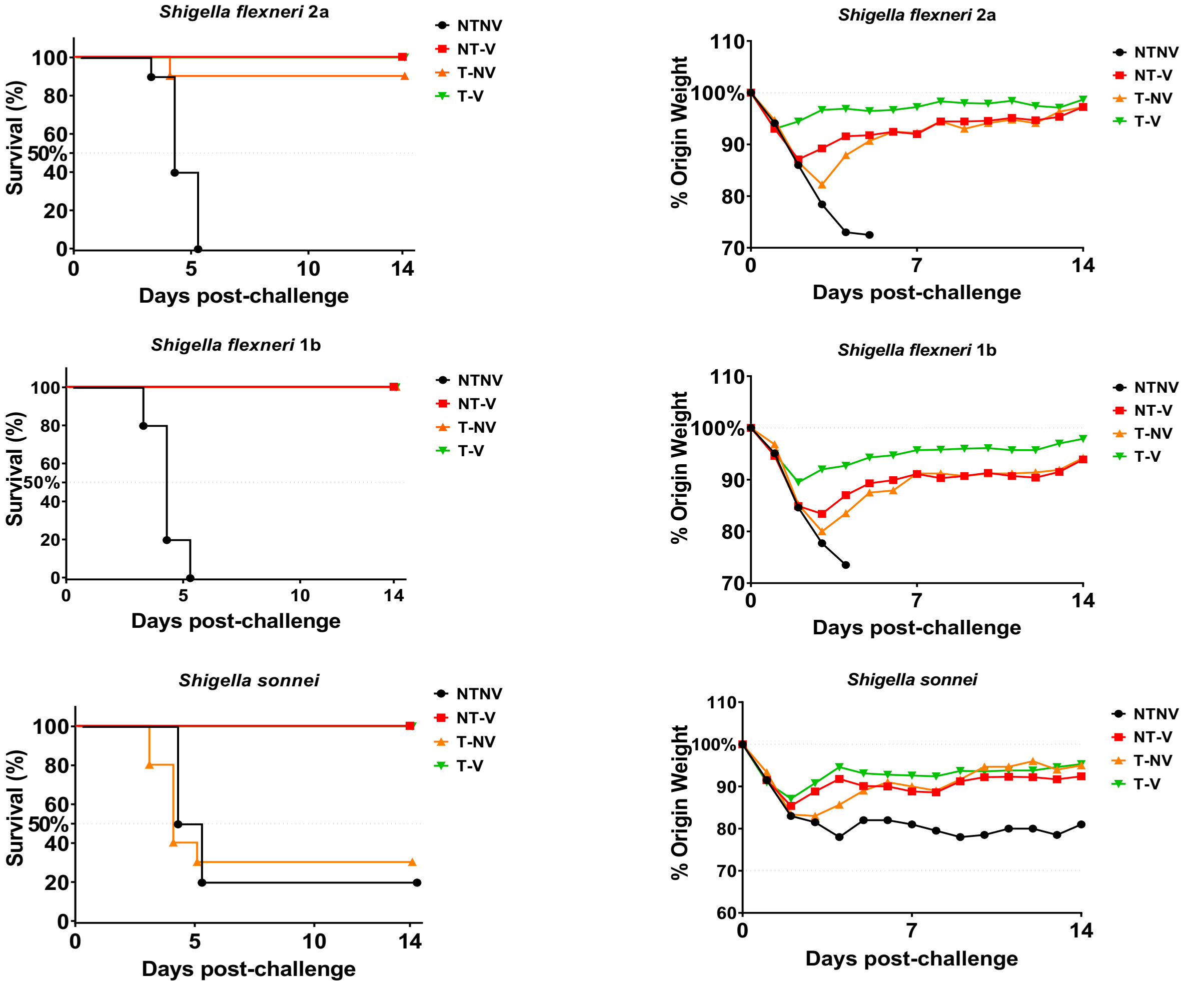

Supplemental Figure 4. Protective efficacy of L-DBF against the *Shigella* lethal challenge in mice. Balb/C mice (n=10) were pre-exposed to two sublethal doses of *S. flexneri* 2a on days -56 and -28 and then vaccinated intranasally (IN) on days 0, 14 and 28 with 25 μg L-DBF. On day 56, the mice were challenged IN with *S. flexneri* 2a, *S. flexneri* 1b or *S. sonnei*.

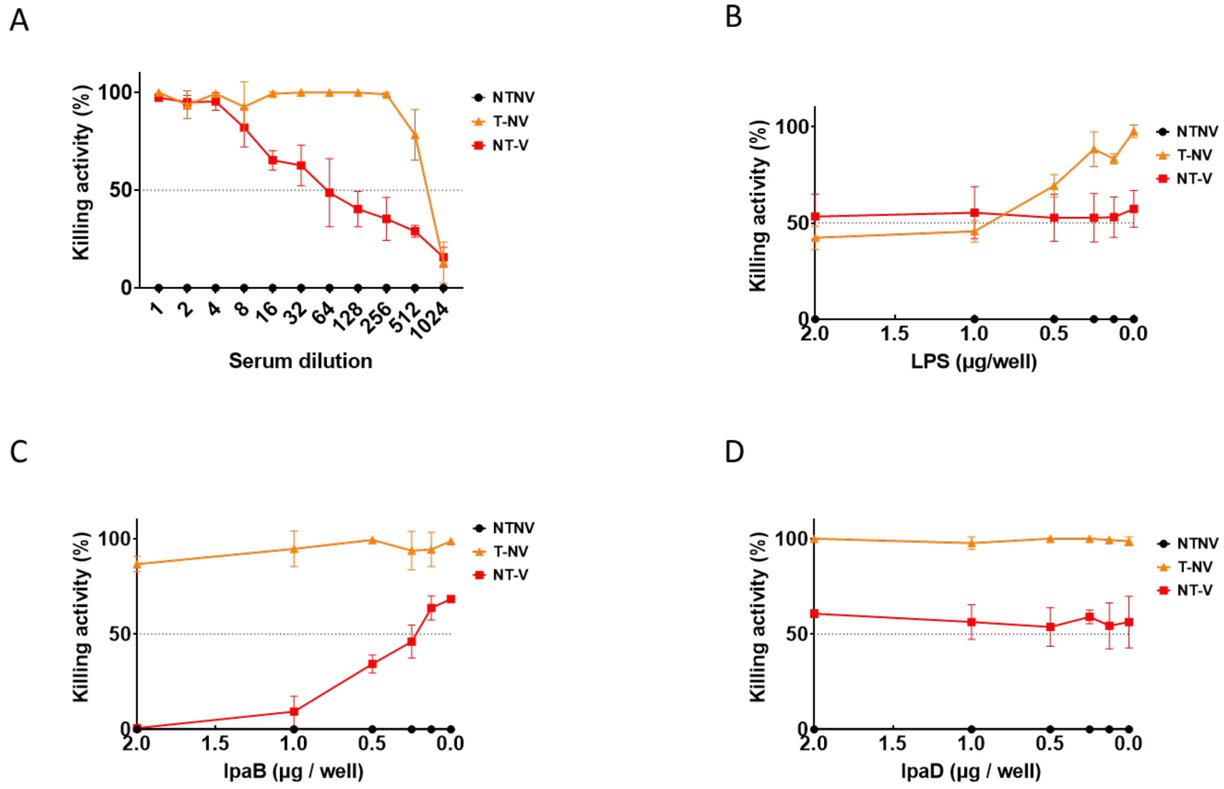

Supplemental Figure 5. Serum bacteriocidal activity (SBA) activity for serum from mice after a single *S. flexneri* 2a pre-exposure. The killing activity (%) in serially diluted pooled serum from NT-NV, T-NV or the NT-V group is shown (**Panel A**). The killing activity (%) in serum from the T-NV group (1:512 dilution) or from NT-V groups (1:64) in competition with serially diluted LPS is shown in **Panel B**. The killing activity (%) in serum from the T-NV group (1:512 dilution) or from the NT-V group (1:8 dilution) in competition with serially diluted IpaB (**Panel C**) or IpaD (**Panel D**) is also shown. Dashed horizontal lines indicate the 50% killing point. CFU counts obtained from wells incubated with serum from the NT-NV group was used as 0% killing (baseline control). Killing activity (%) = (Spots in NT-NV well – spots in test well) / Spots in NT-NV well]. Duplicate experiments with triplicate wells for each test point. Mann-Whitney test was used for analysis. *p<0.05; **p<0.01; ***p< 0.001

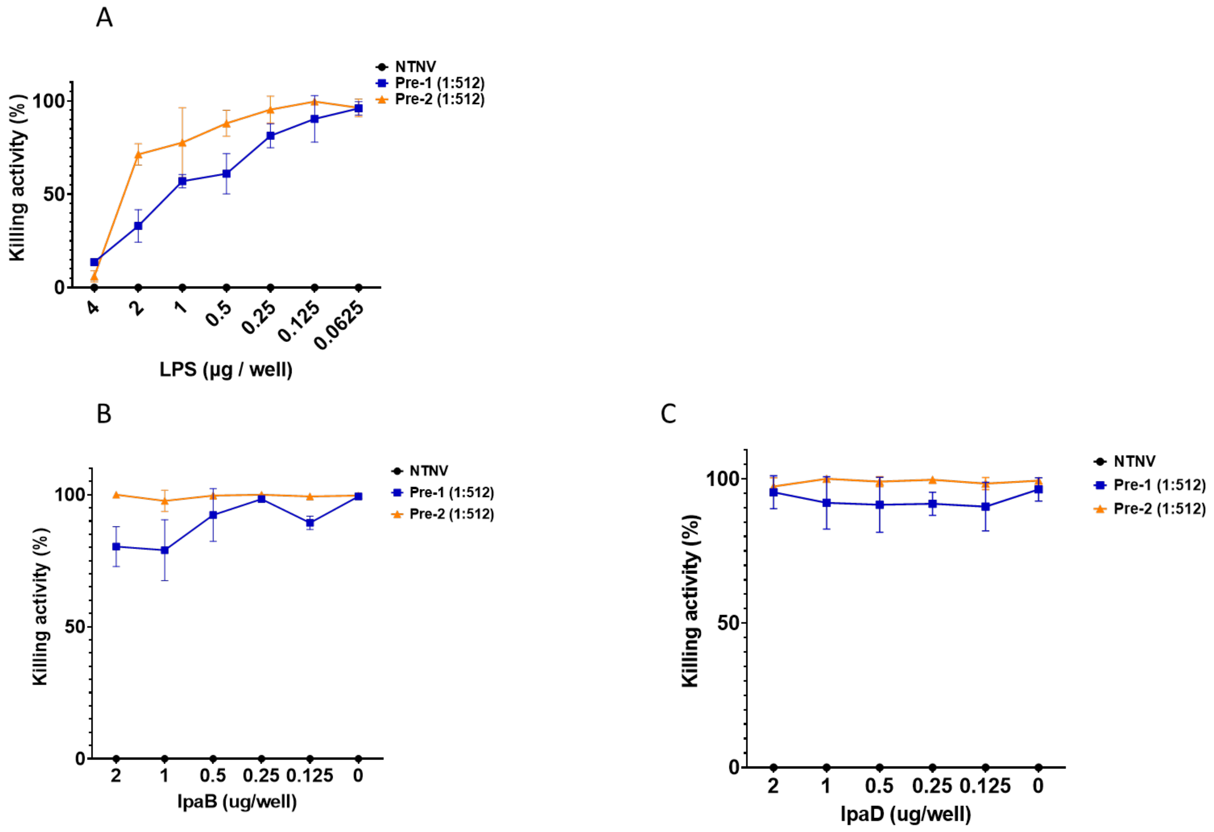

Supplemental Figure 6. Competitive SBA of the samples from mice receiving two sublethal pre-infections with exogenously added *S. flexneri* 2a LPS, IpaB or IpaD. The killing activity (*y* axis) after incubating with pre-1 (serum collected on day 28 after first pre-infection) or pre-2 (serum collected on day 14 after second pre-infection) in the presence of serially diluted LPS (**Panel A**) or IpaB (**Panel B**) or IpaD (**Panels C**) are shown. CFU counts obtained with wells incubated with serum from the NT-NV group was used as 0% killing (baseline). Killing % = (Spots in NT-NV well – spots in test well) / Spots in NT-NV well]. Duplicate experiments with triplicate wells for each test point. Mann-Whitney test was used for analysis. *p<0.05; **p<0.01; ***p< 0.001

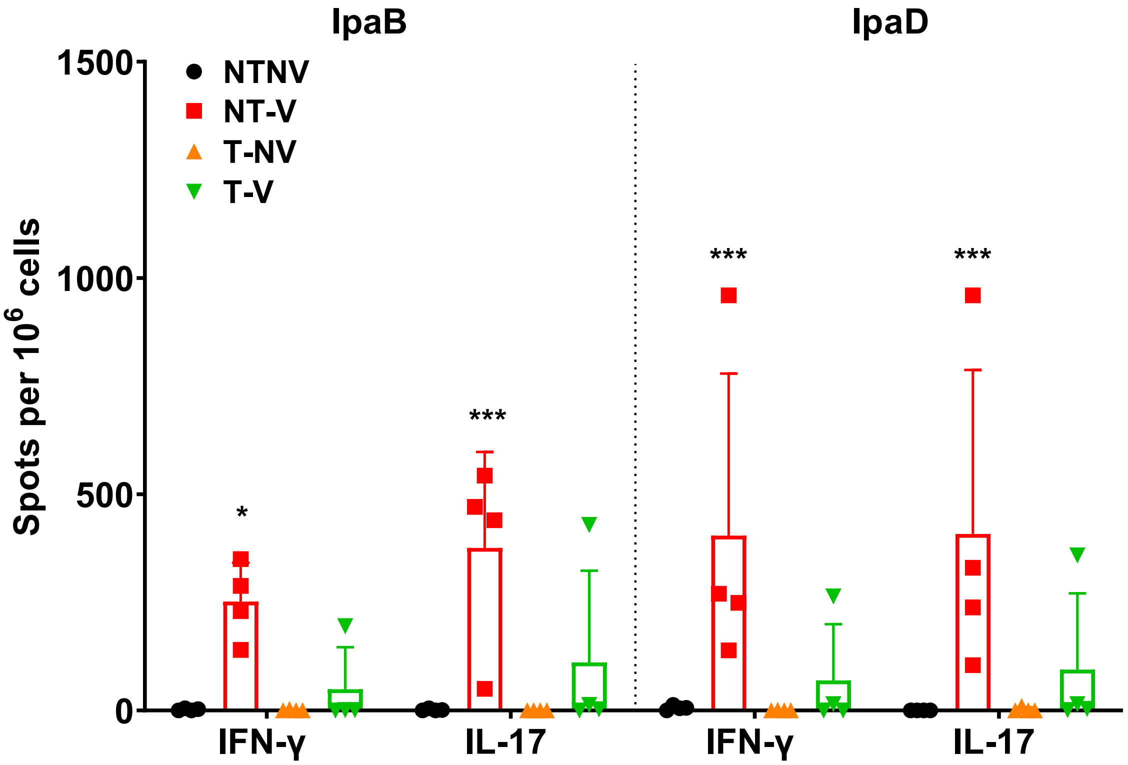

Supplemental Figure 7. The frequency of IL-17 and IFN-γ secreting cells after antigen-specific stimulation. The splenocytes were used to enumerate the antigen-specific IFN-γ and IL-17 secreting cells. Cells were incubated with 10 µg IpaB (**left**) or IpaD (**right**). IFN-γ and IL-17 secreting cells were enumerated by ELISpot analysis and are presented here as spot-forming cells/10^6 cells. The data are plotted as means ± SD for individual mice in each group. Significance was calculated by comparing groups that were unvaccinated (PBS) and mice vaccinated with antigens using two-way ANOVA. *$p<0.05$; **$p<0.01$; ***$p< 0.001$.

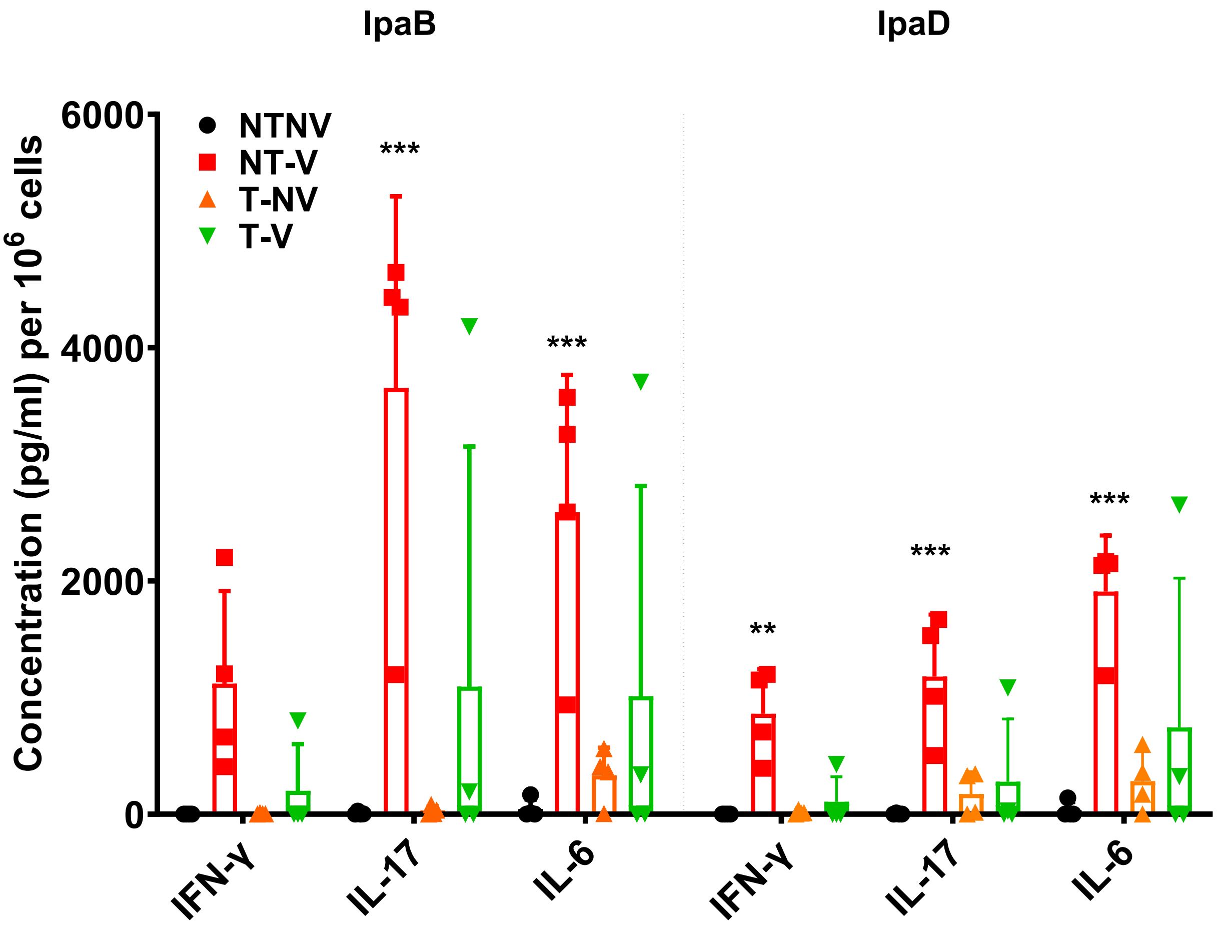

Supp. Figure 8. Concentrations of T cell-related cytokines secreted from splenocytes was determined after stimulation with IpaB or IpaD. Single spleen cell suspensions were used to assess antigen-specific IFN-ɣ, IL-17 and IL-6 secretion. Cells were incubated with 10 µg IpaB (**left**) or IpaD (**right**). Cytokine concentrations were determined by Meso Scale Discovery analysis as per the manufacturer's specifications and are presented here as pg/ml/10^6 cells. The data are plotted as means ± SD for individual mice in each group. Significance was calculated by comparing groups that were unvaccinated (PBS) and mice vaccinated with antigens using two-way ANOVA. *$p<0.05$; **$p<0.01$; ***$p< 0.001$.

Supplemental Table 1: Statistic analysis for the data shown in Supplementary Figure 5A.

Dilution	P Value		
	T-NV vs. NT-V	T-NV vs. NT-NV	NT-V vs. NT-NV
1	0.861 ns	<.001 ***	<.001 ***
2	0.963 ns	<.001 ***	<.001 ***
4	0.716 ns	<.001 ***	<.001 ***
8	0.101 ns	<.001 ***	<.001 ***
16	<.001 ***	<.001 ***	<.001 ***
32	<.001 ***	<.001 ***	<.001 ***
64	<.001 ***	<.001 ***	<.001 ***
128	<.001 ***	<.001 ***	<.001 ***
256	<.001 ***	<.001 ***	<.001 ***
512	<.001 ***	<.001 ***	<.001 ***
1024	0.861 ns	0.035 *	0.009 ***

* Mann-Whitney test. *p<0.05; **p<0.01; ***p< 0.001

Supplemental Table 2: Statistical analysis for the data shown in Figure 2A.

Dilution	P Value		
	Pre-1 vs. Pre-2	NT-NV vs. Pre-1	NT-NV vs. Pre-2
64	0.181 ns	<.001 ***	<.001 ***
128	>0.999 ns	<.001 ***	<.001 ***
256	0.123 ns	<.001 ***	<.001 ***
512	0.106 ns	<.001 ***	<.001 ***
1024	0.002 **	<.001 ***	<.001 ***
2048	0.002 **	<.001 ***	<.001 ***
4096	0.012 *	0.009 **	<.001 ***
8192	0.010 *	0.956 ns	0.174 ns

* Mann-Whitney test. *p<0.05; **p<0.01; ***p< 0.001

Supplemental Table 3: Statistic analysis for the data shown in Figure 2B.

Dilution	P Value					
	NT-NV vs. NT-V	NT-NV vs. T-NV	NT-NV vs. T-V	NT-V vs. T-NV	NT-V vs. T-V	T-NV vs. T-V
16	0.002 **	0.002 **	0.002 **	0.002 **	0.002 **	>.999 ns
32	0.002 **	0.002 **	0.002 **	0.002 **	0.002 **	>.999 ns
64	0.002 **	0.002 **	0.002 **	0.002 **	0.002 **	>.999 ns
128	0.002 **	0.002 **	0.002 **	0.002 **	0.002 **	0.455 ns
256	0.002 **	0.002 **	0.002 **	0.002 **	0.002 **	>.999 ns
512	0.002 **	0.002 **	0.002 **	0.002 **	0.002 **	0.197 ns
1024	0.002 **	0.002 **	0.002 **	0.002 **	0.006 **	0.143 ns
2048	0.048 *	0.002 **	0.061 ns	0.004 **	0.223 ns	0.126 ns
4096	0.182 ns	0.002 **	0.061 ns	0.002 **	0.167 ns	0.108 ns
8192	>.999 ns	0.002 **	0.002 **	0.002 **	0.002 **	0.032 *

* Mann-Whitney test. *p<0.05; **p<0.01; ***p< 0.001

Supplemental Table 4: Statistic analysis for the data shown in Figure 2C.

Dilution	P Value					
	NT-NV vs. NT-V	NT-NV vs. T-NV	NTNV vs. T-V	NT-V vs. T-NV	NT-V vs. T-V	T-NV vs. T-V
16	<.001 ***	<.001 ***	<.001 ***	<.001 ***	<.001 ***	>.999 ns
32	<.001 ***	<.001 ***	<.001 ***	<.001 ***	<.001 ***	>.999 ns
64	<.001 ***	<.001 ***	<.001 ***	<.001 ***	<.001 ***	>.999 ns
128	<.001 ***	<.001 ***	<.001 ***	<.001 ***	<.001 ***	>.999 ns
256	<.001 ***	<.001 ***	<.001 ***	<.001 ***	<.001 ***	>.999 ns
512	<.001 ***	<.001 ***	<.001 ***	<.001 ***	<.001 ***	>.999 ns
1024	<.001 ***	<.001 ***	<.001 ***	<.001 ***	<.001 ***	0.015 *
2048	<.001 ***	<.001 ***	<.001 ***	<.001 ***	<.001 ***	0.002 **
4096	>.999 ns	<.001 ***	<.001 ***	<.001 ***	<.001 ***	0.004**
8192	>.999 ns	<.001 ***	>.999 ns	<.001 ***	>.999 ns	0.002 **

* Mann-Whitney test. *p<0.05; **p<0.01; ***p< 0.001

Supplemental Table 5: Statistic analysis for the data shown in Figure 3A.

Dilution	P Value					
	NT-NV vs. NT-V	NT-NV vs. T-NV	NT-NV vs. T-V	NT-V vs. T-NV	NT-V vs. T-V	T-NV vs. T-V
4	0.081 ns	0.807 ns	0.045 *	0.920 ns	0.656 ns	0.289 ns
2	0.021 *	<.001 ***	<.001 ***	0.091 ns	0.702 ns	0.563 ns
1	0.011 *	<.001 ***	<.001 ***	<.001 ***	<.001 ***	0.893 ns
0.5	0.021 *	<.001 ***	<.001 ***	<.001 ***	<.001 ***	0.195 ns
0.25	0.002 **	<.001 ***	<.001 ***	<.001 ***	<.001 ***	>.999 ns
0.125	<.001 ***	<.001 ***	<.001 ***	<.001 ***	<.001 ***	0.385 ns
0.0625	0.002 **	<.001 ***	<.001 ***	<.001 ***	<.001 ***	0.999 ns

* Mann-Whitney test. *p<0.05; **p<0.01; ***p< 0.001

Supplemental Table 6: Statistic analysis for the data shown in Figure 3B.

Dilution	P Value					
	NTNV vs. NT-V	NTNV vs. T-NV	NTNV vs. T-V	NT-V vs. T-NV	NT-V vs. T-V	T-NV vs. T-V
4	0.002 **	0.002 **	0.002 **	0.008 **	0.002 **	0.129 ns
2	0.002 **	0.002 **	0.002 **	0.002 **	0.023 *	0.002 **
1	0.002 **	0.002 **	0.002 **	0.002 **	0.006 **	0.002 **
0.5	0.060 ns	0.002 **	0.002 **	0.002 **	0.006 **	0.002 **
0.25	0.015 *	0.002 **	0.002 **	0.002 **	0.004 **	0.002 **
0.125	0.002 **	0.002 **	0.002 **	0.002 **	0.002 **	0.002 **
0.0625	0.002 **	0.002 **	0.002 **	0.002 **	0.008 **	0.002 **

* Mann-Whitney test. *p<0.05; **p<0.01; ***p< 0.00

Supplemental Table 7: Statistic analysis for the data shown in Figure 4A.

Dilution	P Value					
	NT-V vs. T-NV	NT-V vs. T-V	NT-V vs. NT-NV	T-NV vs. T-V	T-NV vs. NT-NV	T-V vs. NT-NV
2	<.001 ***	<.001 ***	0.393 ns	>.999 ns	<.001 ***	<.001 ***
1	<.001 ***	<.001 ***	<.001 ***	>.999 ns	<.001 ***	<.001 ***
0.5	<.001 ***	<.001 ***	<.001 ***	>.999 ns	<.001 ***	<.001 ***
0.25	<.001 ***	<.001 ***	<.001 ***	>.999 ns	<.001 ***	<.001 ***
0.125	<.001 ***	<.001 ***	<.001 ***	0.923 ns	<.001 ***	<.001 ***
0	0.003 **	0.001 ***	<.001 ***	0.991 ns	<.001 ***	<.001 ***

* Mann-Whitney test. *p<0.05; **p<0.01; ***p< 0.001

Supplemental Table 8: Statistic analysis for the data shown in Figure 4B.

Dilution	P Value					
	NT-V vs. T-NV	NT-V vs. T-V	NT-V vs. NT-NV	T-NV vs. T-V	T-NV vs. NT-NV	T-V vs. NT-NV
2	<.001 ***	<.001 ***	<.001 ***	>.999 ns	<.001 ***	<.001 ***
1	<.001 ***	<.001 ***	<.001 ***	>.999 ns	<.001 ***	<.001 ***
0.5	<.001 ***	<.001 ***	<.001 ***	>.999 ns	<.001 ***	<.001 ***
0.25	<.001 ***	<.001 ***	<.001 ***	>.999 ns	<.001 ***	<.001 ***
0.125	<.001 ***	<.001 ***	<.001 ***	>.999 ns	<.001 ***	<.001 ***
0	0.001 ***	<.001 ***	<.001 ***	0.998 ns	<.001 ***	<.001 ***

* Mann-Whitney test. *p<0.05; **p<0.01; ***p< 0.001

Supplemental Table 9: Statistic analysis of Figure 4C.

Dilution	P Value					
	NT-V vs. T-NV	NT-V vs. T-V	NT-V vs. NT-NV	T-NV vs. T-V	T-NV vs. NT-NV	T-V vs. NT-NV
2	<.001 ***	<.001 ***	<.001 ***	>.999 ns	<.001 ***	<.001 ***
1	<.001 ***	<.001 ***	<.001 ***	>.999 ns	<.001 ***	<.001 ***
0.5	<.001 ***	<.001 ***	<.001 ***	>.999 ns	<.001 ***	<.001 ***
0.25	<.001 ***	<.001 ***	<.001 ***	>.999 ns	<.001 ***	<.001 ***
0.125	<.001 ***	<.001 ***	<.001 ***	0.879 ns	<.001 ***	<.001 ***
0	<.001 ***	0.002 ***	<.001 ***	0.754 ns	<.001 ***	<.001 ***

* Mann-Whitney test. *p<0.05; **p<0.01; ***p< 0.001

Supplemental Table 10: Statistic analysis for the data shown in Figure 4D.

Dilution	P Value					
	NT-V vs. T-NV	NT-V vs. T-V	NT-V vs. NTNV	T-NV vs. T-V	T-NV vs. NTNV	T-V vs. NTNV
2	<.001 ***	<.001 ***	<.001 ***	0.99 ns	<.001 ***	<.001 ***
1	<.001 ***	<.001 ***	<.001 ***	0.996 ns	<.001 ***	<.001 ***
0.5	<.001 ***	<.001 ***	<.001 ***	0.675 ns	<.001 ***	<.001 ***
0.25	<.001 ***	<.001 ***	<.001 ***	0.982 ns	<.001 ***	<.001 ***
0.125	<.001 ***	<.001 ***	<.001 ***	0.952 ns	<.001 ***	<.001 ***
0	<.001 ***	<.001 ***	<.001 ***	0.999 ns	<.001 ***	<.001 ***

* Mann-Whitney test. *p<0.05; **p<0.01; ***p< 0.001
